# Investigations on the Role of Support and Synthesis Procedure for the Ru_x_–Cu_y_/CNT‐Catalysed Hydrogenolysis of Glycerol to 1,2‐Propanediol

**DOI:** 10.1002/open.70262

**Published:** 2026-07-14

**Authors:** Dominique Lumpp, Samrin Shaikh, Jan‐Dominik Krueger, Fabian Riebesehl, Christiane Roller, Charlotte Ruhmlieb, Baldur Schroeter, Carina Hedrich, Martin Ritter, Irina Smirnova, Bodo Fiedler, Jakob Albert

**Affiliations:** ^1^ Institute of Technical and Macromolecular Chemistry University of Hamburg Hamburg Germany; ^2^ Institute of Polymers and Composites Hamburg University of Technology Hamburg Germany; ^3^ Institute of Physical Chemistry University of Hamburg Hamburg Germany; ^4^ Institute of Thermal Separation Processes Hamburg University of Technology Hamburg Germany; ^5^ United Nations University Hub on Engineering to Face Climate Change at the Hamburg University of Technology United Nations University Institute for Water, Environment and Health (UNU‐INWEH) Hamburg Germany; ^6^ Electron Microscopy Unit Hamburg University of Technology (TUHH) Hamburg Germany

**Keywords:** 1,2‐propanediol, carbon nanotubes, glycerol hydrogenolysis, heterogeneous catalysis, structure‐activity‐selectivity‐relationship

## Abstract

Tailoring support effects plays an important role for controlling activity and selectivity in heterogeneously catalyzed gas–liquid reactions. Sophisticated carbon supports like single‐ and multiwall carbon nanotubes (CNTs) as well as in house produced Globugraphite were used for the synthesis of bimetallic Ru–Cu catalysts, which were tested in a multibatch reactor setup for the chemical hydrogenolysis of glycerol to 1,2‐propanediol (1,2‐PDO) in aqueous solution. In‐depth characterization using multiple tools like inductively coupled plasma optical emission spectrometry, CHNS‐O analysis, N_2_‐physisorption, scanning electron microscopy, transmission electron microscopy, energy‐dispersive X‐ray spectroscopy, powder X‐ray diffraction, H_2_‐temperature programmed reduction, laser diffraction, and point of zero charge measurements revealed the dominating effects that the various carbon supports exert on the catalytic performance. The best‐performing catalyst synthesized by wetness impregnation consisted of Ru–Cu_3_ nanoparticles (NPs) from mixed RuCl_3_ and Cu(NO_3_)_2_ precursors dispersed on multiwall CNT Baytubes with a productivity of *p *= 12.74 g_1,2‐PDO_ g_active_ _metal_^−1^ h^−1^. Its superior activity combined with high 1,2‐PDO selectivity (*S* = 75%) can be explained both by the nature of the highly dispersed Ru and Cu NPs showing the lowest reduction peak temperature on entangled tubes of CNT agglomerates demonstrating a high mesopore volume for optimal reactant accessibility and the highest specific surface area of all investigated materials. This lays the foundation for implementing this highly active and selective catalyst in a future SMART reactor for glycerol valorisation.

## Graphical abstract

Sophisticated carbon supports like single‐ and multiwall carbon nanotubes as well as Globugraphite were used for the synthesis of bimetallic Ru–Cu catalysts, which were tested in a multi batch reactor setup for the chemical hydrogenolysis of glycerol to 1,2‐propanediol (1,2‐PDO) at 220 °C under 30 bar of hydrogen pressure in aqueous solution.

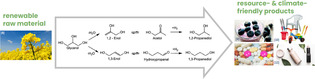

**Abbreviations**
1,2‐PDO: 1,2‐propanediol
BMSSG: ball mill solid state grinding
*c*
: concentration
CD: chemical deposition
CP: coprecipitation
DP: deposition precipitation
EG: ethylene glycol
EtOH: ethanol
HA: hydroxyaceton
IWI: incipient wetness impregnation
*m*
: mass
MWCNT: multiwall carbon nanotube
*n*
: amount of substance
n‐Pr: n‐propanol
SSG: solid state grinding
SWCNT: singlewall carbon nanotube
WI: wetness impregnation

## 1 Introduction

Since its discovery by Radushkevich and Lukyanovich in 1952 [1] and its boost in popularity by the report of Iijima in 1991 [2], carbon nanotubes (CNTs) have become one of the most famous nanotechnologies. CNTs provide useful properties such as high electrical conductivity [3], mechanical [4, 5] and thermal stability [6], resistance against poison effects [7, 8], flexible and high surface area [9], possibility of shaping the morphology during the synthesis process [10, 11] and to change the chemical composition with doping [12, 13, 14]. Moreover, the ability to fine tune the metal–support interaction and flexibility of functionalizing the surface groups to change hydrophilicity, pH, electron donor–acceptor properties and chemical properties of the surface make CNTs a material with a wide range of applications [15].

CNTs are being applied in various fields such as polymer reinforcement, energy and gas storage materials, sensors, electronics, and catalysts [16]. In 1994, CNTs were first used in catalysis when Planeix et al. impregnated CNTs with Ru for the hydrogenation of cinnamaldehyde to hydro‐cinnamyl alcohol [17]. Since then, CNTs have been utilized extensively as a support in heterogeneous catalysis and can be functionalized for that purpose in a variety of ways. These techniques can be divided into the following four different categories: i) nanoparticle (NP) dispersion on functionalized CNTs, ii) physicochemical approaches, iii) electrochemical deposition, and iv) electroless deposition. Those synthesis methods all offer different advantages for catalyst preparation [18].

Functionalized CNTs have a wide variety of applications in heterogeneous catalysis. For instance, they were employed in several CO/H_2_ conversion processes, such as higher alcohol synthesis [19], methanol synthesis [20], and Fischer–Tropsch synthesis [16, 21]. Bai et al. showed that the use of CNTs instead of SiO_2_ improved the reducibility of the active Co phase, thereby enhancing the activity and stability of the catalyst [21], while Tavasoli et al. observed a similar promotion in comparison to Al_2_O_3_‐supported catalysts [22]. Different noble metals supported on CNTs, like Pt [23, 24], Ru [24, 25], or Cu [24, 26], were used for the catalytic oxidation of aniline and phenol. In ammonia synthesis, Ru supported on active carbon is an alternative to replace the industrially used Fe‐based catalysts; however, those catalysts showed in several studies a loss of activity due to sintering after a prolonged reaction time [27]. Instead, using CNTs as a support leads to a higher thermal stability of the catalyst [28]. Bychko et al. showed a Ni/CNT catalyst performing the hydrogenation of ethylene and its reverse reaction, the dehydrogenation of ethane in the gas‐phase and the liquid‐phase hydrogenation of *p*‐nitrotoluene and *α*‐methylstyrene, showing the wide applicability of their catalyst in different hydrogenation/dehydrogenation reactions [29].

Additionally, CNT‐supported catalysts have been applied for the hydrogenolysis of glycerol [30, 31]. This reaction in particular is of great interest, as the production of sustainable glycerol has increased in tandem with the current growth in biodiesel production, making it more attractive for new applications, like 1,2‐propanediol (1,2‐PDO), which can be obtained through the hydrogenolysis of glycerol and is used as a monomer for the polymer industry, pharmaceuticals, cosmetics, food and animal feed industry [32, 33, 34, 35, 36]. Suarez et al. studied Ru‐based catalysts on different support materials, like active carbon, CNTs and zeolites for 1,2‐PDO synthesis. They found that the electron donor character of the CNTs stabilizes electron‐rich Ru NPs favouring formation of 1,2‐PDO on metal sites but simultaneously enhances the successive C—C single bond cleavage of glycerol, compared to the active‐carbon‐supported catalysts [37]. Li et al. synthesized a stable and reusable Ru–Fe/CNT catalyst which achieved full glycerol conversion and a combined selectivity of over 75% to 1,2‐PDO and ethylene glycol. A synergetic effect between the Ru–Fe bimetallic NPs and the interaction of those with the CNTs was responsible for the high catalytic activity [38]. Wu et al. used a Ru–Cu catalyst supported on MWCNTs produced by the chemical replacement method for glycerol hydrogenolysis. They concluded that the deposition of the Ru particles on the surface of the Cu particles promoted the hydrogen activation of the Cu metal, resulting in higher 1,2‐PDO yields [30]. Liu et al. developed bimetallic Ru–Cu catalysts, which were impregnated on different support materials, like SiO_2_, TiO_2_, Al_2_O_3_, and ZrO_2_. The ZrO_2_‐supported catalyst showed the best results under harsh reaction conditions of 100 bar H_2_ pressure and 180 °C, where full glycerol conversion and a selectivity to 1,2‐PDO of 78% were achieved [39]. The mechanism analysis of Gatti et al. showed that in a range of *T* = 250 °C–350 °C and *p *= 40–100 bar the hydrogenation reaction of acetol to 1,2‐PDO is limited by thermodynamic equilibrium. For the glycols, the formation of 1,2‐PDO is favoured over 1,3‐PDO; while the formation of iso‐propanol is favoured over that of 1‐propanol [40]. Sherbi et al. used an improved wetness impregnation approach to synthesize their CNT‐supported Ru–Cu catalyst. They could observe a promotion effect by the surface interaction of Ru and Cu leading to a preference of Cu‐promoted C—O bond cleavage over the Ru‐promoted C—C cleavage [31]. In previous studies, we further optimized this catalyst system by investigating different combinations of transition metals, like Cu, Fe, Co, and Ni, and noble metals such as Ru, Pt, Pd, Ir, Au, and Ag. Furthermore, we tried to statistically evaluate these results by a mathematical model to further improve the hydrogenolysis catalyst. It was observed that the combination of Ru and Cu is indeed the most active and selective catalyst for the 1,2‐PDO production from glycerol [41]. Hassenstein et al. used this catalyst to establish a kinetic model, where they found out that the formation of ethylene glycol could be efficiently suppressed by separating both reaction steps from each other by operating under different gas atmospheres in the same setup, leading to a tremendous increase in 1,2‐PDO yield [42, 43].

This work investigates the role of the CNT support for the hydrogenolysis of glycerol to 1,2‐PDO by connecting catalytic activity studies with various structural analysis techniques after examining the role of the active metals [41] and the kinetics of the reaction [42]. Therefore, we systematically synthesized different Ru–Cu catalysts on various singlewall (SW) as well as multiwall (MW) CNTs and in‐house synthesized Globugraphite. Furthermore, the ratio between the active metals Ru and Cu was varied to find the optimal metal configuration for maximal 1,2‐PDO yield. Hence, we tested different metal precursors, as the location and distance between the metal particles, as well as the counter ions can impact the catalysts performance. While the ingredients for the catalyst synthesis are important, the way the material is synthesised is also an essential parameter in catalyst development. Therefore, different synthesis procedures were tested and compared. Different wetness impregnation methods like classical wetness and incipient wetness impregnation were tested and compared to different precipitation approaches such as chemical deposition, deposition precipitation, and coprecipitation. With the aspect to sustainability solvent‐free mechanical impregnation methods have been tested as well.

## 2 Experimental Details

### 2.1 Materials

The glycerol used for the experiments was purchased from Alfa Aesar with a purity of 99+% and used without further purification. NC7000 were purchased from Nanocyl, Baytubes from Bayer MaterialScience AG, Flycarbon from Carbon Fly and TuBalls from OCSiAl. For the catalyst synthesis, the following metal precursors were used: RuCl_3_*·3H_2_O (Sigma–Aldrich, 38%–42% Ru), Ru(NO_3_)_3_ (Thermo Fisher, 31,3% Ru), Ruthenium(III)acetate (Apollo Scientific, 99%), Ruthenium(III)acetylacetonate (Merck, 99%), Cu(II)(NO_3_)_2_*·3H_2_O (Sigma–Aldrich, 99%), CuCl_2_*·2H_2_O (Alfa Aesar, 99%), Copper(II)acetate (Sigma–Aldrich, 98%), Copper(II)acetylacetonate (Sigma–Aldrich, 99,9%), and NaBH_4_ (Arcos Organic, 98%). For the manufacturing of Globugraphite, ZnO powder (Reagent Pulus, Sigma–Aldrich) with a particle size of <5 µm and purity of 99.9%) is mixed with 45 vol% of polyvinyl butyral (PVB) (Mowital B 60HH, Kuraray) with a purity of 97.5%. In the CVD‐synthesis toluene (ChemSolute) with a purity of 99.9% is used as a carbon source.

### 2.2 Synthesis of Globugraphite Support

The synthesis of Globugraphite is based on a two‐step process already described by Marx et al. [44]. In the first step, a ceramic template was manufactured by combining zinc oxide and PVB powder, mixing in an attritor with deionized water at 625 rpm for 1 h. The mixture was dried at 55 °C for 5 days and sieved before pressing a green body in a uniaxial press. A cylindrical shape with a diameter of 9.3 mm and a weight of 250 mg was chosen. The green bodies were then sintered in a Nabertherm L9/11 furnace with a heating rate of 120 °C/h to 400 °C on an aluminium oxide plate.

For the chemical vapor deposition step of the synthesis, a Carbolite HZS 12/−900 tubular reactor was used. Argon was used as inert gas with a flow of 0.2 L/min. The reactor was heated to 760 °C, when the reaction temperature was reached a hydrogen flow of 60 mL/min was supplied and 5 mL/h of the precursor toluene was preheated to 200 °C and added. In this step the template morphology was replicated with carbon. After 2 h, the precursor flow was stopped and the reactor heated to 900 °C and held at the temperature for 2 h to ensure the reduction of the zinc oxide. Afterwards the reactor was cooled under an increased argon flow of 0.4 l/min. Ten templates were used per synthesis. Resulting from this manufacturing route is a carbon structure with a hierarchical pore structure and a 3D, globular morphology.

### 2.3 Catalyst Preparation

A total of seven different synthesis methods were carried out, which are described in detail in the following part. Usually, 2 g of supported catalyst were synthesized.

#### 2.3.1 Wetness Impregnation

The applied wetness impregnation method has already been described in a previous study by Lumpp et al. [41]. 1.9 g of the CNTs were added in a round bottom flask. First of all 89.5 mg (0.342 mmol, 1.00 equiv.) ruthenium(III) chloride trihydrate and 248 mg (1.03 mmol, 3.01 equiv.) copper(II) nitrate trihydrate were dissolved in 100 mL water each and the precursor solutions were added one after another in the round‐bottom flask. The catalyst solution was heated to 80 °C and stirred at 110 rpm using a rotary evaporator for 5 h. After that, the liquid was removed. The catalyst was mortared carefully and dried in an oven (Nabertherm L9/11) for 8 h at 110 °C.

#### 2.3.2 Incipient Wetness Impregnation

First, 89.5 mg (0.342 mmol, 1.00 equiv.) ruthenium(III) chloride trihydrate was suspended with 248 mg (1.03 mmol, 3.01 equiv.) copper(II) nitrate trihydrate in 2.28 mL of the used solvent. The amount of water needed was based on the pore volume of the support, which was determined via N_2_‐physisorption. Subsequently the solution was added to 1.90 g of the support material and the suspension was stirred at room temperature for 2 h using a stirring plate. Finally, the synthesized catalyst was dried at 110 °C for 8 h.

#### 2.3.3 Chemical Deposition

The synthesis was carried out similarly to the method presented by Wu et al. [45]. Initially, 249 mg (1.03 mmol, 2.99 equiv.) of copper(II) nitrate trihydrate was dissolved in 300 mL of demineralized water with 1.90 g of Baytubes. Subsequently, 89.9 mg (0.344 mmol, 1.00 equiv.) of ruthenium(III) chloride trihydrate was dissolved in 60 mL of demineralized water and added to the first solution. The reaction solution was stirred for 2 h at 0 °C. Then, 100 mL of 0.1 M NaBH4 solution was slowly added at 0 °C, and the reaction mixture was stirred for an additional 2 h. Finally, the synthesized catalyst was filtered, washed with demineralized water, and dried at 110 °C for 8 h.

#### 2.3.4 Deposition Precipitation

The synthesis was performed similarly to the method described by Liu et al. [46]. First, 50 mL of a 0.05 M NaOH solution was prepared, to which 1.90 g of Baytubes was added. The suspension was stirred at room temperature for 30 min. Then, a solution of 89.6 mg (0.343 mmol, 1.00 equiv.) ruthenium(III) chloride trihydrate and 249 mg (1.03 mmol, 3.01 equiv.) of copper(II) nitrate trihydrate in 40 mL of demineralized water was added to the suspension. The reaction solution was stirred for another 2 h at room temperature and then allowed to mature for 68 h. In the final step, the synthesized catalyst was filtered, washed with demineralized water, and dried at 110 °C for 8 h.

#### 2.3.5 Coprecipitation

The synthesis was based on the method presented by Liu et al. [46]. Initially, 89.6 mg (0.343 mmol, 1.00 equiv.) of ruthenium(III) chloride trihydrate was dissolved with 251 mg (1.04 mmol, 3.03 equiv.) of copper(II) nitrate trihydrate and 1.90 g of Baytubes in 40 mL of demineralized water. Then, 40 mL of 0.5 M NaOH solution was added, and the mixture was stirred for 12 h at room temperature. The solution was then allowed to mature for 24 h. Finally, the catalyst was filtered, washed with demineralized water, and dried at 110 °C for 8 h.

#### 2.3.6 Solid‐State Grinding in a Mortar

Initially, 91.2 mg (0.349 mmol, 1.00 equiv.) of ruthenium(III) chloride trihydrate, 251 mg (1.04 mmol, 2.98 equiv.) of copper(II) nitrate trihydrate, and 1.90 g of Baytubes were weighed. The solid mixture was then ground using a mortar and pestle for 30 min.

#### 2.3.7 Solid‐State Grinding Using a Ball Mill

First, 89.6 mg (0.343 mmol, 1.00 equiv.) of ruthenium(III) chloride trihydrate was weighed and mixed with 248 mg (1.03 mmol, 3.01 equiv.) of copper(II) nitrate trihydrate and 1.90 g of Baytubes. The 2 L mill was filled with 2.5 kg of steel balls with a diameter of 15 mm. The mixture was then placed into a ball mill and milled for 30 min at 238 rpm.

### 2.4 Analytics for Catalyst Characterization

#### 2.4.1 Inductively Coupled Plasma Optical Emission Spectrometry

The sample preparation is described in the ESI. The elemental concentrations of the following elements were determined by inductively coupled plasma optical emission spectrometry (ICP‐OES) (Perkin Elmer, Avio 550): Al (analytical wavelength: 396.153 nm), Fe (238.204 nm), Cu (327.393 nm), Ru (240.272 nm), and Co (228.616 nm). Fe, Co, and Al are common impurities in CNTs as those metals are usually used as catalysts in the CNT production. To check for reproducibility and accuracy, individual sample splits were measured three times and checked against quality control standards with known certified concentrations. The concentrations of the samples were determined via external calibration against standards with known concentrations. Blanks were usually negligible but were subtracted from the individual sample element concentrations if significant. The quality control standards were usually between 90% and 110%. If significant outliers were detected, measurements were repeated.

#### 2.4.2 Organic Elemental Analysis (CHNS‐O)

CHNS was measured using the “Unicube” measuring device from the company Elementar. The sample was burned at 1150 °C with the addition of O_2_. The gases produced were passed through WO_3_ and Cu using He as a carrier gas, producing N_2_, CO_2_, H_2_O, and SO_2_. CO_2_, H_2_O, and SO_2_ were retained on an adsorption column, while the nitrogen content was measured using a thermal conductivity detector (TCD). The other gases were released individually by heating and also measured. CO_2_ and H_2_O were measured using a TCD, SO_2_ using an IR detector. The measurements were calibrated with sulfanilamide and acetanilide.

Oxygen was measured using the “Oxycube” measuring device from the company Elementar. The sample was pyrolyzed at 1450 °C over coal dust, and the CO produced was measured using a TCD. The measurements were calibrated with benzoic acid and acetanilide.

#### 2.4.3 N_2_‐Physisorption

Low‐temperature N_2_ adsorption–desorption analysis was used (Nova 3000e Surface Area Analyzer, Quantachrome Instruments) to determine the mass specific surface area (*S*_m_), mesopore size distribution, and mesopore volume (*V*_meso_) of the synthesized catalysts. An overall sample mass of ≈20 mg was used for each analysis. Samples were degassed under vacuum at a temperature of 100 °C for 6 h prior to analysis. *S*_m_ was estimated using the Brunauer–Emmett–Teller (BET) method based on highly correlated linear fitting (Figures S9 and S10, Table S3) of the BET model, (*p*/*p*_0_ range = 0.025–0.31) derived from Type IV N_2_ adsorption isotherms. *V*_meso_ and the mean mesopore diameter (*d*_pore,mean_) were estimated via the BJH (Barrett–Joyner–Halenda) method applied to the desorption branch of the isotherms. QSDFT analysis was performed using the equilibrium model with a cylindrical pore geometry assumption. Micropore surface area and micropore volume were estimated using the t‐plot method (Figures S13 and S14, Table S3) based on the Harkins–Jura thickness equation.

#### 2.4.4 Powder X‐Ray Diffraction

Crystal structure determination was performed through powder X‐ray diffraction (PXRD) using a PANalytical MDP X’Pert Pro diffractometer, operating in Bragg–Brentano geometry with Cu Kα radiation (*λ* = 0.1541 nm). The diffraction angle was measured over a range of 5° to 90°, with a sampling rate of 0.013° and a time per step of 72.42 ms.

#### 2.4.5 Scanning Electron Microscope and Energy‐Dispersive X‐Ray

Scanning electron microscopy (SEM) imaging with various magnifications was done at 5 kV acceleration voltage using a LEO Gemini 1550 (Zeiss) equipped with an Inlens detector and ESB detector. The energy‐dispersive X‐ray (EDX) data were recorded within the same setup at 20 kV using the EDX detector Ultim Max 100 (Oxford Instruments). An integration time of 80 s was set for all EDX measurements on several areas of each sample, respectively. The software AzTec (Oxford Instruments) was used for data evaluation.

#### 2.4.6 High‐Resolution Transmission Electron Microscopy and Energy‐Dispersive X‐Ray mapping

For high‐resolution images of the samples  a JEM‐2200FS (JEOL) TEM operating at 200 kV was used. EDX mapping was performed in STEM mode using an Oxford X‐Max 100 TLE detector.

#### 2.4.7 H_2_‐Temperature Programmed Reduction

Temperature programmed reduction (TPR) using H_2_ was measured using a ChemBET Pulsar (Fa. Quantachrome Instruments). Prior, the samples (0.05 g) were exposed to a N_2_ gas flow (80 mL/min) and heated up to 200 °C (20 °C/min) for 10 min to remove surface H_2_O, followed by cooling down to 40 °C. The sample was heated up again under H_2_/N_2_ (5/95 v/v) gas flow (80 mL/min, 30 °C/min) to 850 °C. The H_2_ uptake was measured by a TCD.

#### 2.4.8 Point of Zero Charge

The point of zero charge (PZC) measurements are based on the method developed by Wesner et al. [47]. Therefore, 40 mL solutions of sodium nitrate with a concentration of 0.1 M were prepared, the pH was adjusted using sodium hydroxide solution and nitric acid. The pH was set to values ranging from 2 to 11 to create 10 solutions with varying pH levels (pH_initial_). These solutions were then treated with the carbon support (200 mg). The suspensions were stirred for 24 h at 300 rpm on magnetic stirrer plates. Afterwards, the suspensions were filtered, and the pH of the filtrate was measured again (pH_final_). To determine the PZC, the ΔpH (pH_final − pH_initial) of each solution was calculated and plotted against the initial pH value of the solution (Table S2). The linear region of the curve was fitted, and the intersection point, where ΔpH = 0) was determined as the PZC. The plots can be seen in Figure S2.

#### 2.4.9 Laser Diffraction

Laser diffraction was used to investigate a possible change in particle size using a HELOS (H3641) from Sympatec using a laser and lens setup to detect particles between 0.5 and 200 μm.

### 2.5 Catalytic Testing

Before the catalytic hydrogenolysis reaction, the synthesized catalysts were reduced in a tube furnace (Nabertherm R50/250/12) using forming gas with 5 vol% hydrogen in nitrogen at 550 °C for 8 h with a heating rate of 120 °C h^−1^ and a gas flow of 50 L h^−1^. 550 °C as reduction temperature was used based on previous studies [31]. A calcination step was not performed because the precursors are removed during reduction and the material is therefore exposed to less thermal stress, which could affect the particle size through agglomeration. The hydrogenolysis reaction was carried out in a 4‐fold multibatch reactor setup using high‐pressure stainless‐steel reactors (material number: 1.4571) with a reaction volume of 21 mL (Figure S1). Normally, 100 mg of catalyst was used. In addition to the catalyst, 10 g of a substrate solution consisting of 20 wt‐% glycerol and demineralized water was added to the reactor, as well as a stirring bar made of teflon. The reactors were closed and placed on the heating blocks made of aluminium on a stirring plate and connected to rupture discs and the gas inlet. The stirrer was set to 300 rpm and the reactors were then flushed three times with nitrogen and a pressure of 30 bar was built up for the pressure test. If the pressure remained constant over 1 h, the pressure was released. The reactors were then flushed twice with hydrogen and then pressurized with hydrogen to 30 bar, so the pressure reached a value of 50 bar at the desired reaction temperature of 220 °C. After the reaction temperature was reached, the stirrer speed was set to 1000 rpm in order to start the gas entrainment and thus the reaction. After 20 h, the reaction was stopped by switching off the heat supply and the reactors were cooled down to room temperature. After the reactors reached room temperature, gas samples were taken from the reactor using gas bags. The catalyst was separated by filtration. The liquid phase was analysed via high‐performance liquid chromatography (HPLC) and ^1^H and ^13^C nuclear magnetic resonance (NMR) spectra to confirm the identity of the products.

### 2.6 Analytics for Catalytic Testing

#### 2.6.1 High‐Performance Liquid Chromatography

The liquid phase collected after the reaction was analysed by HPLC for various products. For this purpose, each sample has been filtered (0.45 μm) prior to analysis. The used HPLC is a Nexera 40 from SHIMADZU with a polymer phase organic acid column, 300 mm × 8 mm separation from the company Chromatographie‐Service GmbH. The eluent used was a sulphuric acid aqueous solution with a concentration of 4 mmol/L at a flow rate of 0.8 mL min^−1^ at 25 °C. After the column, the components were detected by a refractive index detector. The detected products were calibrated. A chromatogram containing all the possible compounds of the reaction is shown in Figure S25 whereas Table S6 shows the measured retention times. Figure S26 shows the calibration of the detected compounds.

#### 2.6.2 Gas Chromatography

The gas samples were analysed with a Varian 450‐GC equipped with a Shin‐Carbon‐ST‐Column of 2 m × 0.75 mm. The samples were injected through a 250 μL sample tube and were passed through the column in a stationary phase. The temperature program includes an initial temperature of 40 °C, for 2.5 min. After that, the column is heated to 250 °C in 11.5 min. This temperature is kept constant for additional 12 min. The front column pressure was set to 70 psi, the one in the middle to 10 psi. The products were analysed by a TCD with a setpoint of 300 °C and a flame ionization detector (FID) that is set to 200 °C. The detectable products have been calibrated. Example chromatograms of the TCD and FID are shown in Figures S27 and S28.

#### 2.6.3 Nuclear Magnetic Resonance Spectroscopy

The liquid samples have been analysed qualitatively by using ^1^H and ^13^C NMR. All NMR spectra were measured with a Bruker AVANCE III HD 400 MHz. The samples were prepared as follows: 600 µL of the filtered reaction solution and 100 µL D_2_O as deuterated solvent have been filled in an NMR tube. The NMR analysis was done with the software MestReNova.

### 2.7 Calculations

The following calculations have been carried out in order to evaluate the catalytic performance of the synthesized catalyst materials.

The glycerol conversion was calculated according to Equation (1).

(1) $$X=\frac{c_{0,\text{Glycerol}}-c_{\text{Glycerol}}}{c_{0,\text{Glycerol}}}\times 100\%$$

The yields of both liquid‐ and gas‐phase products were calculated by Equation (2).

(2) $$Y_{i}=\frac{c_{i}}{c_{0,\text{Glycerol}}}\times 100\%$$

The selectivity to the desired product 1,2‐PDO was calculated by Equation (3).

(3) $$S_{i}=\frac{Y_{i}}{X}\times 100\%$$

In order to fully reveal the reaction mechanism, the carbon balance for each experiment was calculated by Equation (4).

(4) $$\text{Carbon balance}=\frac{\sum n_{i}\times \text{number of carbons}}{n_{\text{Glycerol}}\times 3}\times 100\%$$

The productivity of the catalysts as an evaluation criterion was determined using Equation (5).

(5) $$P_{\mathrm{cat}}=\frac{m_{1,2-\mathrm{PDO}}}{m_{\text{active metal}}*t_{R}}$$

## 3 Results and Discussion

### 3.1 Characterization of the Synthesized Catalysts

Building on previous results using NC7000 as a CNT support [31, 41], we further investigated the role of various CNT supports in this study. Therefore, we compared four different commercially available single‐ and multiwalled CNTs, shown in Table 1. Additionally, we synthesized Globugraphite as a reference carbon material [48].

**TABLE 1:** Overview of the commercial CNTs used in this study.

Name	Manufacturer	Application
NC7000	Nanocyl SA, Belgium	Material science and energy sector
Baytubes	Bayer Material Science AG, Germany	Material science and medicine
Flycarbon	Carbon Fly, Japan	Mobility, aerospace, and life science
TuBalls	OCSiAl, Luxembourg	Polymers and electrodes

In the first step, we investigated the elemental composition of the different carbon materials using ICP‐OES for the analytics of the inorganic elements and CHNS‐O for the organic composition.

Table 2 shows the ICP‐OES results of the different carbon supports used in this study. The highest amount of impurities was found in Flycarbon with 16.9% Co and 9.8% Fe. These impurities are residues from the industrial production process, were Fe, Co, and Al‐based catalysts were used [49]. 14.7 wt.% of Fe could be found in the TuBalls, while the NC7000 showed a contamination of 5.6 wt.% of Al. The purest CNTs are the Baytubes, which only show small contamination of Co and Al with 0.29 and 0.18 wt.%, respectively. No impurities could be found in the synthesized Globugraphite (for details see corresponding section in ESI).

**TABLE 2:** ICP‐OES results of the investigated carbon materials.

Support	Co/g/Kg	Ru/g/Kg	Fe/g/Kg	Al/g/Kg
NC7000 [41]	2.1	<2.5	5.0	56.0
Baytubes	2.9	<2.5	<2.5	1.83
Flycarbon	169	<9.62	98	<3.0
TuBalls	<0.5	<0.5	147	<3.0
Globugraphite [48]	<0.5	<0.080	<0.5	<3.0

The results of the organic elemental analysis for the different carbon supports are shown in Table 3. Flycarbon exhibits the highest purity by showing a carbon content of over 99%, combined with low amounts of N and O, indicating a low number of functional groups and therefore less defects. Compared to that, the Baytubes have a slightly higher content of N and O and hence a lower carbon content of almost 97.4%. In contrast to this, the previously discussed NC7000 shows a quite significant drop in carbon content with 88.2%. This can be explained by the high amount of O with almost 5% and the previously discussed high Al ratio of 5.6%. The lowest C content can be found in the TuBalls with 84.9%. At the same time, they contain the highest amount of N and S of all investigated CNTs. Responsible for the low carbon share are the high Fe impurities. While the amount of Globugraphite was only sufficient for one measurement, a carbon ratio of 93.3% was observed, while almost no other organic components were detected. Both methods combined are able to completely explain the composition of the CNTs.

**TABLE 3:** Results of the CHNS‐O analysis of the used carbon materials.

Support	C/%	H/%	N/%	S/%	O/%	Sum/%
NC7000	88.22 ± 0.07	0.72 ± 0.03	0.25 ± 0.01	0.01 ± 0.00	4.90 ± 0.21	94.09 ± 0.31
Baytubes	97.40 ± 0.09	0.60 ± 0.03	0.23 ± 0.01	0.02 ± 0.00	0.92 ± 0.00	99.16 ± 0.14
Flycarbon	99.54 ± 0.12	0.64 ± 0.01	0.14 ± 0.14	0.02 ± 0.00	0.18 ± 0.04	100.51 ± 0.39
TuBalls	84.94 ± 0.50	0.57 ± 0.02	0.32 ± 0.02	0.47 ± 0.01	1.38 ± 0.17	87.67 ± 0.72
Globugraphite	93.3	0.14	0.09	0.21	0	93.74

After taking a look into the elemental composition of the support materials, we synthesized a different series of Ru–Cu impregnated carbon catalysts using the procedures described in the Experimental Section. All catalysts were synthesized using the wetness impregnation approach, apart from the catalysts of the synthesis variation. Herein, all synthesized catalysts used in this study were analysed for their elemental composition using ICP‐OES. Taking a look at the ICP‐OES results of the catalysts supported on various carbon supports shown in Table S1, we observed that for the NC7000‐supported catalyst the desired loading was achieved. With a metal loading of 4.43% the desired value was also achieved for the Ru–Cu_3_/Flycarbon catalyst. For the catalysts supported on Baytubes this wasn’t the case, as the total metal loading was just 2.78%, as both the Ru and Cu loading were lower than desired. The TuBall‐supported catalyst showed a Ru loading of 1.53%, which is close to the desired 1.74%, but the Cu loading was found to be lower than the detection limit, showing that the synthesis procedure wasn’t suited for this kind of CNT. With 3.42% the loading of the Globugraphite supported catalyst was also slightly lower than expected, which is a result of the lower Cu loading. For the catalysts in which the ratio between Ru and Cu was varied and the Baytubes were used as a support, the previous behaviour was observed again, as the total metal loading varied in a range between 2.78% and 4.95%. Nevertheless, the ratio between the metals could be varied successfully. When comparing the different metal precursors used in the synthesis, we could emphasize that by using nitrate‐based precursors the closest loadings to the desired 5% with 3.9% could be achieved. This catalyst had the highest Ru loading of 1.55% in the precursor variation. Through the use of chlorides, acetates, and acetylacetonates, catalysts with loadings of 2.98%, 3.30%, and 2.65%, respectively, could be synthesized. Hereby the nitrate‐based catalyst had the highest Cu ratio, resulting in the overall highest loading. Additionally, we could observe very similar loadings for the catalysts, when the synthesis method was changed. The overall loading was in a range of 3.71% to 4.30%. The two mortared catalysts had a loading of 4.27%, resp. 1.35%. This could be explained by the mechanical stress on the CNTs exerted by the ball mill, which is detrimental for metal impregnation.

**FIGURE 1:**
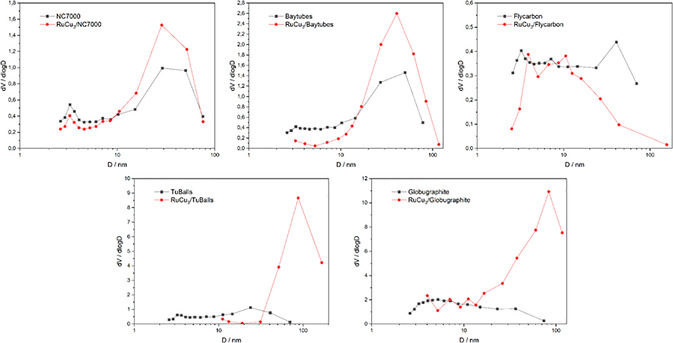
Pore size distribution of the different carbon support materials and synthesized Ru–Cu catalysts.

Table 4 summarizes the microstructural properties of the various CNT supports, which were investigated via N_2_‐physisorption. The carbon supports exhibit distinct mesoporous architectures that strongly influence their behaviour upon Ru and Cu loading. The N_2_ adsorption–desorption isotherms reveal predominantly mesoporous characteristics for all supports [50] (Figure S3), although differences in adsorption behaviour were observed between various materials. In particular, NC7000, Baytubes, Flycarbon and Globugraphite display type IV(a)‐like features, whereas TuBalls deviate from this behaviour, consistent with its negative BET C constant. Despite broadly similar pore size distribution (PSD) shapes, the supports differ substantially in accessible mesoporosity. NC7000 and Baytubes feature wide mesopore populations extending into the low‐macroporous regime (Figure 1), most likely reflecting interbundle voids characteristic of entangled MWCNT networks. Flycarbon, although structurally comparable in PSD shape, exhibits a markedly reduced mesopore volume and surface area. Its distribution mirrors that of NC7000 and Baytubes but appears at substantially lower intensity, indicating fewer accessible mesopores rather than a fundamentally different pore width distribution. TuBalls, composed of SWCNT bundles, possess a higher fraction of smaller mesopores, while Globugraphite exhibits a hierarchical pore architecture comprising mesopores and small macropores.

**TABLE 4:** Physicochemical properties of the neat CNTs and metal impregnated CNT catalysts.

Catalyst	aSpecific surface area, m^2^ g^−1^	bMesopore volume, cc/g	bMean pore diameter, nm	cMetal dispersion, %	cParticle diameter, nm	Point of zero charge
NC7000 [41]	252	0.919	14.8	—	—	6.72
Baytubes	249	1.202	19.5	—	—	7.88
Flycarbon	176	0.539	12.1	—	—	7.17
TuBalls	513	0.983	8.6	—	—	7.38
Globugraphite	404	2.001	17.6	—	—	6.67
Ru–Cu_3_/NC7000 [41]	232	1.140	19.9	52.82	2.512 ± 0.75	—
Ru–Cu_3_/Baytubes	198	1.534	27.4	25.05	5.297 ± 2.15	—
Ru–Cu_3_/Flycarbon	86	0.351	15.1	70.36	1.886 ± 0.13	—
Ru–Cu_3_/TuBalls	1666	4.264	12.4	20.69	6.412 ± 1.91	—
Ru–Cu_3_/Globugraphite	1354	7.011	4.0	52.30	2.537 ± 0.75	—

a Determined with N_2_‐physisorption using the BET method.

b Determined with N_2_‐physisorption using the BJH method.

c Determined with HR‐TEM [38].

After impregnation with Ru and Cu, the textural response differs markedly (Figures S5 and S7, Table S3) between the supports. For NC7000 and Baytubes, the specific surface area decreases moderately, while the mesopore volume slightly increases. In contrast, Flycarbon exhibits a decrease in both surface area and mesopore volume. These changes occur despite low total metal loadings (∼2–5 wt%), which are insufficient to account for the observed textural variations either through simple mass dilution effects or through the additional surface area theoretically provided by the metal NPs. A more detailed discussion of the N_2_‐physisorption results can be found in the ESI.

In addition to pore structure, surface chemical properties may also influence metal deposition and particle growth. The PZC of the supports lies between 6.67 and 7.88 (Figure S2), indicating comparable surface charge characteristics under the applied preparation conditions. The metal dispersion of the catalysts was determined by HR‐TEM and can be seen in Table 4 and lies in a range between 20% and 70%, with the Flycarbon‐supported catalyst having the highest dispersion of 70%. Interestingly, Ru–Cu_3_/Flycarbon exhibits the highest metal dispersion despite having the lowest BET surface area among the investigated catalysts. This indicates that metal dispersion is not directly correlated with the N_2_‐accessible surface area. Instead, the comparatively limited mesoporosity may restrict particle growth, and residual Fe/Co impurities present in Flycarbon could act as nucleation sites during deposition. These interpretations remain mechanistic hypotheses consistent with the observed structural trends. The calculation was done according to Li et al. [38], including the volume of a Ru atom, the molecular weight of Ru, its density, the surface area of a Ru atom, and the particle diameter. Regarding the average particle size all supported catalysts match with the dispersion nicely, as the catalysts with the highest dispersion possess the smallest particle diameter.

**FIGURE 2:**
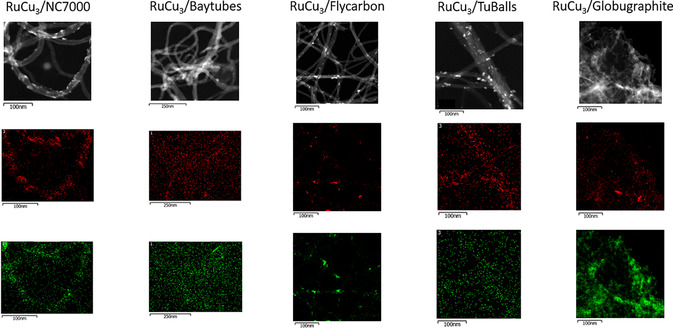
HAADF–STEM images and TEM‐EDX mapping of the Ru–Cu_3_ catalysts supported on the different carbon materials.

The analysis of the morphology of the support materials and the synthesized catalysts by SEM, transmission electron microscopy (TEM), high‐angle annular dark‐field (HAADF) imaging, and EDX mapping are shown in Figures S16–S22. The HAADF–STEM imaging and EDX mapping of Ru and Cu in the different supported Ru–Cu_3_‐catalysts (Figure 2), showed that both metals are located on the surface of the tubes in small crystals. For NC7000, Baytubes, and TuBalls, the Ru and Cu particles are highly dispersed, while Flycarbon shows smaller agglomerates for both metals. Globugraphite shows those agglomerated particles just for Ru. We could also confirm that the CNT structure remains intact after it was exposed to the synthesis conditions, as seen in Figures S20 and S21.

Using TEM‐EDX mapping of the synthesized catalysts for the metal precursor variation before and after the reduction are shown in Figures S16 and S17. We observed that both metals are well distributed regardless of the used precursor combination. However, after the reduction of the catalyst, agglomeration of the nitrate‐, chloride‐, and acetylacetonate‐based catalysts could be observed. The different morphology of the CNTs can be seen in the SEM pictures, as shown in Figure S18. The tubes of NC7000 and Baytubes seem to be entangled with each other, forming groups of CNT agglomerates. For the Baytubes, this is a result of the production process, which is following an expanding universe mechanism [51]. The primary catalyst particles are agglomerated and due to the highly porous nature of the carbon support the growth happens in and around the particles. With the growing nanotubes needing more space resulting in the disintegration of the catalyst particles. The Flycarbon seems to have a more carpet‐like structure, with the tubes having a parallel orientation, while the TuBalls form a loose web of tubes. In the TEM pictures of the materials, we can clearly see the remnant impurities of the TuBalls and NC7000, which were found in the ICP‐OES. For the Flycarbon and Baytubes, no metal particles were found in the pictures. For the catalysts of the precursor variation no significant differences could be seen in the TEM pictures, as shown in Figure S22.

The particle size distribution density curve and cumulative distribution of the supports are shown in Figure S15, the average particle sizes can be seen in Table S5. We could observe that the average particle size for NC7000, Baytubes and TuBalls are in a comparable range between 57 and 68 μm. Because of its fibrous structure, this analytic method isn’t suitable for Flycarbon.

Neat CNTs and supported catalysts were further investigated by PXRD. The diffractograms of the neat CNTs are shown in Figure S23. For the three MWCNTs NC7000, Baytubes, and Flycarbon the typical reflexes for CNTs were observed at 26° (002), 43° (110), 54° (004) and 78° (006) [52]. The TuBalls and the Globugraphite are distinctly amorphous. Taking a look at the Ru–Cu catalysts supported on the different carbon supports (Figure S24), reflexes of CuO can be observed at 32.5° (110), 39.7° (022), and 50.2° (202) [53]. Compared to previous studies the copper reflexes can now be observed due to the higher copper ratio in the catalyst [41]. In contrast to that reflexes caused by Ru can’t be observed. Likely, the Ru phases are too small to be observed and are superimposed by the noise of the CNTs. This indicates that the metal is present in small crystals and is highly dispersed on the CNTs.

**FIGURE 3:**
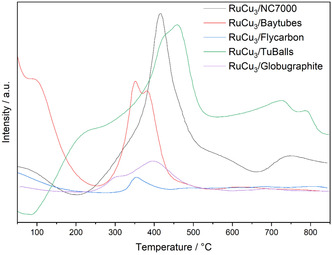
H_2_‐TPRs of the Ru–Cu catalysts supported on the different carbon materials.

The reducibility of the supported catalysts was tested by performing H_2_‐TPR measurements up to a temperature of 850 °C. The results are shown in Figure 3. For most of the catalysts, the reduction peaks of Ru (+3) and Cu (+2) to their metallic states have been merged into a broad peak in the range between 200 °C and 550 °C. An exception to this is the Ru–Cu_3_/Baytubes catalyst, showing a quite significant reduction peak at 100 °C, which can be attributed to the reduction of Ru (+3) to metallic Ru. This indicates, that the Ru‐particles in those catalysts are easier to reduce as reported by Bai et al. [54]. The additional peak at 750 °C for the NC7000‐based catalyst can be attributed to the methanization of the CNT, a process well described in the literature and catalysed by the Ru supported on its surface [55]. Interestingly, the reduction of the SWCNT‐supported catalyst starts at the lowest temperature. It indicates that the metal clusters of this catalyst are smaller compared to the other clusters on the other carbon materials increasing their reducibility. The high peak area is also a sign of a higher hydrogen consumption compared to the other catalysts, which can be explained by the additional reduction of the high Fe impurities found in the ICP‐OES. The same effect can be observed in a less intense form for the NC7000‐based catalyst. The support materials which are characterized by a high degree of purity, Baytubes, Flycarbon, and Globugraphite, show significantly smaller peak areas, as there aren’t any impurities that are being reduced, leading to a higher hydrogen consumption. On top of that these catalysts show reduction peaks that haven’t merged completely, indicating that the reduction takes place in several steps [31].

### 3.2 Catalytic Hydrogenolysis Experiments

**FIGURE 4:**
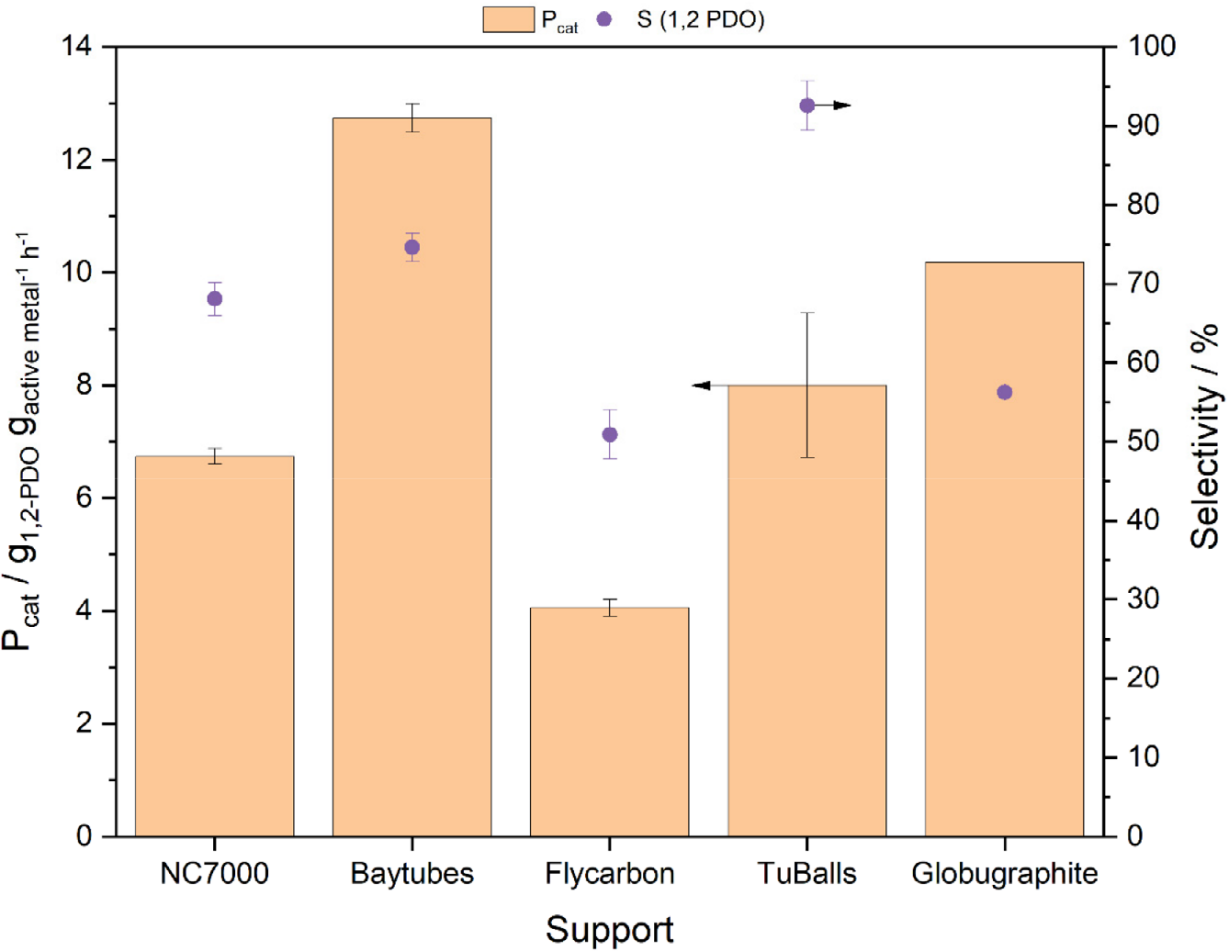
Catalytic performance of the Ru–Cu_3_ catalyst supported on different carbon materials. *m*_Cat_ = 100 mg, loading = 5 wt.%, *T* = 220 °C, *p*_H2_ = 30 bar, *t* = 20 h, *n* = 1000 rpm, *c* (Gly) = 20 wt.%, 10 g reaction mixture.

Based on previous findings using NC7000‐supported Ru–Cu catalysts [32], we further investigated the impact of the CNT support on the results for the glycerol hydrogenolysis to 1,2‐PDO in this study [41]. The results of the hydrogenolysis experiments are shown in detail in Table S7. The results of the control experiments shown in Table S7 demonstrate that the neat CNTs show no activity in the glycerol hydrogenolysis reaction. Consequently, we focussed on Ru–Cu‐based catalysts with a metal ratio of 1:3 supported on different carbon materials, which were synthesised by wetness impregnation. The results of the catalytic experiments of those Ru–Cu_3_ catalysts are shown in Figure 4. The catalyst supported on Baytubes shows the highest productivity of 12.74 g_1,2‐PDO_ g_active_ _metal_^−1^ h^−1^ and a high 1,2‐PDO selectivity of 74.6%, which is an improvement compared to similar catalyst systems reported in literature, as shown in Table S8. The higher productivity can be explained by a combination of different factors. In comparison to the other support materials, a small amount of impurities results in less undesired byproducts being formed during the reaction, while the higher pore volume and average pore size diameter allow an easier diffusion of the reactants towards the active centres. Also, as seen in Figure 2 the Ru and Cu particles are nicely dispersed on the surface of the Baytubes, whereas smaller agglomerates can be seen in the catalysts supported on Flycarbon and Globugraphite. Additionally, the easier Ru reduction is also a factor for the higher catalyst activity, which is further confirmed by the low hydroxyaceton (HA) selectivity, meaning that the formed HA is easily hydrogenated to the desired product. The catalysts supported on Globugraphite and TuBalls show a comparable productivity of 10.18 and 8.0 g_1,2‐PDO_ g_active_ _metal_^−1^ h^−1^, with the TuBall‐supported catalysts having the overall highest selectivity of 92.6%. The high surface areas and pore volumes shown in Table 4 seem to have a beneficial effect on the catalyst activity. However, compared to the Baytubes a smaller average pore diameter could hinder the access of the reactants to the pore system. The NC7000‐supported catalyst shows a productivity of 6.74 g_1,2‐PDO_ g_active_ _metal_^−1^ h^−1^. This can be attributed to the smaller pore volume and the higher ratio of impurities in the CNTs shown in Table 2. The overall lowest productivity was achieved by the catalyst supported on Flycarbon with 4.05 g_1,2‐PDO_ g_active_ _metal_^−1^ h^−1^, which is contributed to its low specific surface area and pore volume. Reproducibility studies, shown in Figure S29, give a standard deviation for the productivity of 0.25 g_1,2‐PDO_ g_active_ _metal_^−1^ h^−1^ and 1.80% for the selectivity. Independently of the support material, the carbon balance of the experiments could be closed. Because of these findings we decided to continue our study using Baytubes as the most promising carbon support.

**FIGURE 5:**
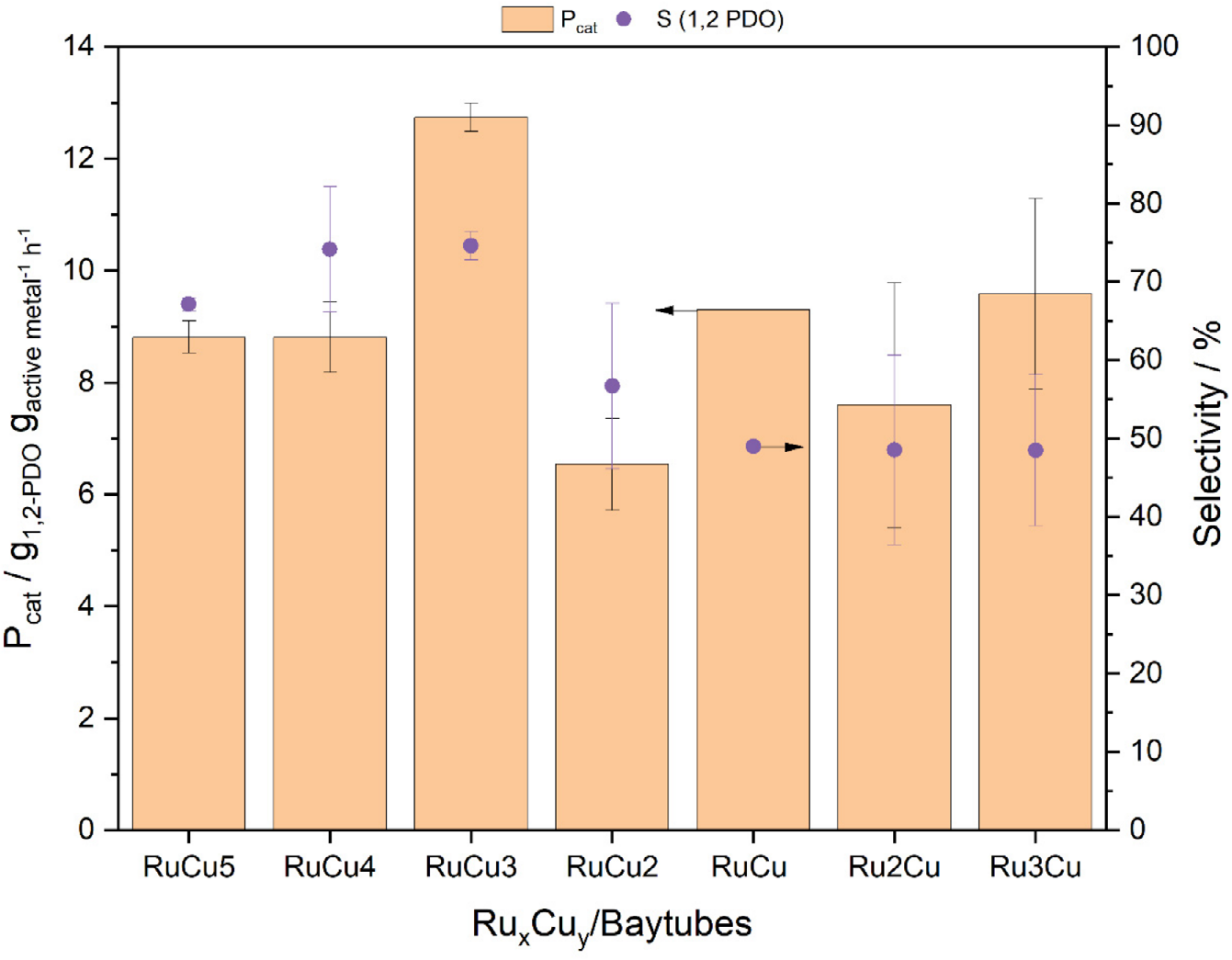
Catalytic performance of the catalysts with different Ru:Cu ratio supported on Baytubes. *m*_Cat_ = 100 mg, loading = 5 wt.%, *T* = 220 °C, p_H2_ = 30 bar, *t* = 20 h, *n* = 1000 rpm, *c* (Gly) = 20 wt.%, 10 g reaction mixture.

In the next step of the catalyst development, the ratio of the active metals Ru and Cu was varied using Baytubes as the most promising CNT support, while keeping the overall metal loading constant, to find the optimal ratio between both active metals. The results are shown in Figure 5.

The outcome of the experiments matches previous studies, as the catalyst with a Ru–Cu ratio of 1:3 is the most active system with a productivity of 12.74 g_1,2‐PDO_ g_active_ _metal_^−1^ h^−1^ [41]. Interestingly, most of the investigated catalysts have a similar productivity in the range of 8.81 and 9.59 g_1,2‐PDO_ g_active_ _metal_^−1^ h^−1^. The two outliers are Ru–Cu_2_ and Ru2–Cu with a productivity of 7.60 and 6.54 g_1,2‐PDO_ g_active_ _metal_^−1^ h^−1^. While the specific surface areas of those catalysts, which can be seen in Table S3 aren’t showing significant differences compared to the more active systems, the metal loadings of those two systems are closer to the desired 5 wt.% than the other catalysts, resulting in a comparatively lower productivity. Even though most of the catalysts show a slightly lower loading, the overall ratio between Ru and Cu is varied between the desired values. We could also observe an increase of the catalyst's selectivity with a rise in Cu ratio. Conversely, a higher Ru ratio results in a catalyst with a higher activity, as seen in Figure S30. These catalysts possess a lower selectivity and tend to form more byproducts, as seen in Table S7. Hence, a maximum in 1,2‐PDO yield is observed in the Ru–Cu_3_/Baytubes‐catalyst, as it unites a relatively speaking high activity with a high product selectivity. Because of its high productivity and selectivity, we focused on a metal ratio of 1:3.

**FIGURE 6:**
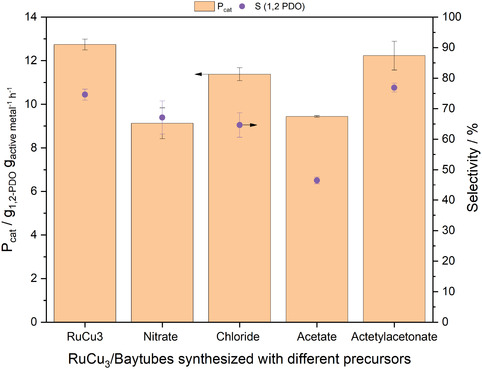
Catalytic performance of different Ru–Cu_3_/Baytubes catalysts, where different precursors have been used. The following precursors were used: For the standard catalyst RuCl_3_ and Cu(NO_3_)_2_; Nitrate: Ru(NO_3_)_3_ and Cu(NO_3_)_2_; Chloride: RuCl_3_ and CuCl_2_; Acetate: Ru(III)acetate and Cu(II) acetate; Acetylacetonate: Ru(III)acetylacetonate and Cu(II)acetylacetonate. *m*_Cat_ = 100 mg, loading = 5 wt.%, *T* = 220 °C, *p*_H2_ = 30 bar, *t* = 20 h, *n* = 1000 rpm, *c* (Gly) = 20 wt.%, 10 g reaction mixture.

Next, we wanted to investigate the influence of the metal precursor on the performance of the catalyst, as the counter ions can have a huge influence on the manner the metals are deposited on the surface. Therefore, we synthesized catalysts with the optimal ratio of Ru and Cu using nitrate‐, chloride‐, acetate‐, and acetylacetonate‐based precursors and compared them to the previous Ru–Cu_3_/Baytubes catalyst, where RuCl_3_ and Cu(NO_3_)_2_ were used as metal precursors. The results of this study are shown in Figure 6. We observed in this study that the choice of the precursor has an impact on the catalyst activity. With a productivity of 12.74 g_1,2‐PDO_ g_active_ _metal_^−1^ h^−1^ the catalyst where RuCl_3_ and Cu(NO_3_)_2_ were used as metal precursors again showed the highest activity. By replacing RuCl_3_ during the catalyst synthesis through Ru(NO_3_)_3_ resulted in a decrease in productivity to 9.13 g_1,2‐PDO_ g_active_ _metal_^−1^ h^−1^ and selectivity to 67.1%. The results of the ICP‐OES elemental analysis in Table S1 show for the nitrate‐based catalyst a slightly higher metal loading of 3.94%; however, the higher loading didn’t result in better performance. If the nitrate‐based precursors were replaced by chloride, the resulting catalyst showed a better activity than the nitrate‐based catalyst with a productivity of 11.38 g_1,2‐PDO_ g_active_ _metal_^−1^ h^−1^ being slightly lower than the catalyst were the mixed precursors were used. This indicates that the chloride of the precursors doesn’t seem to have a negative impact on the catalytic performance, or it is getting removed completely during the reduction process. Using acetates as precursors resulted in the catalyst with the lowest productivity and selectivity. This catalyst showed a high activity, which resulted in the formation of side products like acetol (8%), ethanol (4%), and the consecutive hydrogenolysis product *n*‐propanol (8%). An explanation for the high activity may lie in the high metal dispersion, that was seen in the TEM‐EDX mapping, as shown in Figures S16 and S17. The highest selectivity of 76.9% was observed in the experiments, where the acetylacetonate‐based catalyst was used. On top of that the catalyst possesses a high productivity of 12.12 g_1,2‐PDO_ g_active_ _metal_^−1^ h^−1^. By comparing the specific surface areas of the investigated catalysts (see Tables 4 and S3), we can clearly see that the catalyst with the mixed precursors has the highest specific surface area. Regarding the pore volume, acetate‐ and chloride‐based catalysts show the highest productivities. While the N_2_‐physisorption isotherms of the catalysts show a very similar behaviour (Figure S7), we can clearly see a shift for the mixed precursor catalyst in the PSD in Figure S8. The higher productivity of the mixed catalyst could be explained through the higher presence of mesopores in the range between 10 and 50 nm. The other four catalysts have the majority of their pore volume at a higher diameter resulting in them having a pore system consisting of fewer but larger pores, resulting in an overall similar pore volume.

**FIGURE 7:**
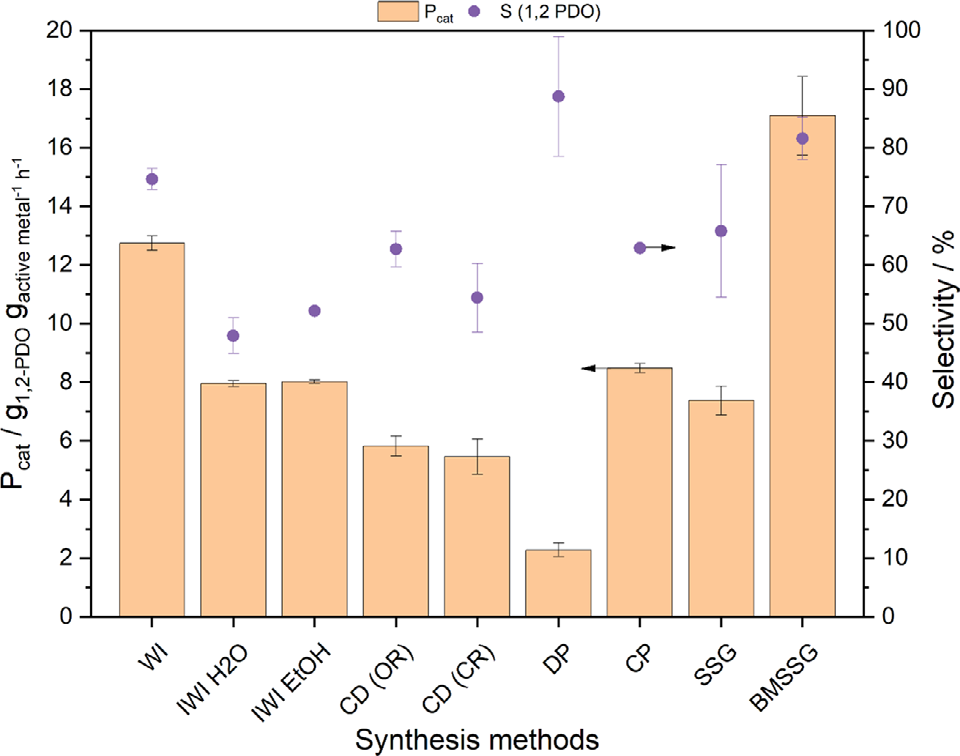
Catalytic performance of different Ru–Cu_3_/Baytubes catalysts, where different precursors have been used. WI = wetness impregnation, IWI = incipient wetness impregnation, CD = chemical deposition, DP = deposition precipitation, CP = co precipitation, SSG = solid state grinding, BMSSG = ball mill solid state grinding. *m*_Cat_ = 100 mg, loading = 5 wt.%, *T* = 220 °C, p_H2_ = 30 bar, *t* = 20 h, *n* = 1000 rpm, *c* (Gly) = 20 wt.%, 10 g reaction mixture.

After investigating the influence of the metal precursor, we wanted to find the best possible synthesis method for the Ru–Cu_3_/Baytubes catalyst, as the manner and method of how the metals are deposited on the CNTs can play a crucial role in their catalytic performance. For this, several catalysts with the previously determined metal ratio and precursor were synthesized. We chose to investigate different wetness impregnation, precipitation, and solvent‐free synthesis methods. A detailed description of those procedures can be found in the Experimental section. The catalytic results are shown in Figures 7 and S31. Because the ICP‐OES results in Table S1 showed big differences in the metal loading of the catalysts, glycerol conversion, 1,2‐PDO yield, and selectivity instead of productivity were used to compare the various catalysts. We could observe that the synthesis method plays a key role in the catalytic performance. The highest 1,2‐PDO yield with 44.1% could be obtained with the catalyst were wetness impregnation was used, followed by the coprecipitation catalyst with 43.7% and the solid‐state grinded catalyst with 38.6% 1,2‐PDO yield. With regard to the glycerol conversion, the best performing catalyst was the system were the incipient wetness impregnation was used with water as a solvent with a conversion of 75.3%, the coprecipitation catalyst with 69.5% and the incipient wetness impregnation catalyst in which ethanol was used with a conversion of 69.3%. Regarding the selectivity, the deposition precipitation catalyst was able to achieve a selectivity of 88.7% followed by the solid state ball mill grinded catalyst with 81.6% and the wetness impregnated catalyst with 74.6%. For the CD catalyst, we could also observe, that a reduction in the oven (OR) in addition to the chemical reduction during the synthesis (CR) improved the yield from 32.7% to 34.9% and the selectivity from 54.4% up to 62.7%, while the conversion decreased from 60.1% to 55.7%. This indicates that even if the catalyst is chemically reduced the reduction isn’t complete and a reduction in the oven is necessary to improve the catalyst performance. As seen in Table S4, the kind of synthesis method has also a major influence on the specific surface area. The catalysts in which incipient wetness impregnation was used have a surface area in the range between 169 and 180 m^2^ g^−1^, while the precipitation‐based catalysts lie in a range between 223 and 235 m^2^ g^−1^. However, a correlation between surface area and catalyst performance could not be observed.

Finally, we wanted to investigate the recyclability of the best performing catalyst. Therefore, the catalyst was separated from the reaction mixture via filtration and washed with water. Afterwards, the catalyst was reduced again and the hydrogenolysis experiment was performed as usual. The results of this study are shown in Figure S32. In the first recycling step, a decrease in productivity from 12.74 to 9.73 g_1,2‐PDO_ g_active_ _metal_^−1^ h^−1^ was observed, further decreasing to 9.06 g_1,2‐PDO_ g_active_ _metal_^−1^ h^−1^ after the second recycling step. Regarding the selectivity, a decrease from 74.61% to 53.88% in the first step and an increase to 66.21% in the second step were observed. This can be explained through changes in the physicochemical properties shown in Table S4, as the specific surface area decreases from 249 to 203 m^2^ g^−1^, while the total pore volume remains almost unchanged after three recycling steps. The decreased surface area can be a result of the repeated reduction of the catalyst, as this additional thermal stress could promote agglomeration and sintering of the metal particles. As no traces of Ru and Cu could be detected in the reaction solutions by ICP‐OES, leaching can be excluded as explanation for the performance loss. We didn’t find traces of Ru and Cu in the reaction solutions; however, the ICP results of the recycled catalyst show a decrease in Cu ratio, as seen in Table S1, while the Ru loading remains unchanged. Therefore, leaching of the Cu particles could explain the loss in performance.

## 4 Conclusions

This study showed a rigorous catalyst optimization study for an efficient CNT‐supported glycerol hydrogenolysis catalyst. The influence of different carbon support materials, metal ratios, and precursors, as well as synthesis procedures on the activity and selectivity of the different Ru–Cu_*x*_/carbon catalysts was investigated. Ru–Cu_3_/Baytubes was observed to be the best‐performing catalyst with a productivity of 12.74 g_1,2‐PDO_ g_active_ _metal_^−1^ h^−1^ and a high 1,2‐PDO selectivity of 74.6% due to its high mesoporous volume allowing for a good accessibility of the reactants in combination with a high metal dispersion on the surface of the entangled tubes with high interbundle voids and the lowest H_2_ reduction peak temperature at 100 °C attributed to the reduction of Ru (+3) to metallic Ru (0). A Ru:Cu metal ratio of 1:3 results in the best performing catalyst as it is the sweet spot between the activity of Ru and the selectivity of Cu. Using the combination of RuCl_3_ and Cu(NO_3_)_2_ as precursors offered an advantageous balance between activity and selectivity, resulting in the highest 1,2‐PDO yield as this combination with mixed Cl^−^ and NO_3_^−^ precursors shows the highest specific surface area. It could be demonstrated that the catalyst synthesis procedure has a significant influence both on the activity and selectivity of the hydrogenolysis reaction, with the wetness impregnation method resulting in the catalyst with the highest presence of mesopores leading to the highest productivity.

## Author Contributions

**Dominique Lumpp:** investigation (catalyst synthesis, catalyst characterisation and catalytic experiments), methodology (catalyst synthesis, characterisation and catalytic experiments), conceptualization, writing original draft. **Samrin Shaikh:** supervision, writing – review & editing. **Jan‐Dominik Krueger:** supervision writing – review & editing. **Fabian Riebesehl:** investigation (catalyst characterisation), methology (catalyst characterisation). **Christiane Roller:** writing original draft, investigation (support synthesis), methology (support synthesis). **Charlotte Ruhmlieb:** investigation (microscopy), methology (microscopy), formal analysis (microscopy). **Baldur Schroeter:** writing original draft, formal analysis (physisorption). **Carina Hedrich:** formal analysis (microscopy). **Martin Ritter:** formal analysis (microscopy). **Irina Smirnova:** writing – review & editing, supervision. **Bodo Fiedler:** writing – review & editing, supervision, conceptualization. **Jakob Albert:** writing – review & editing, supervision, conceptualization, funding acquisition.

## Funding

This study was supported by Deutsche Forschungsgemeinschaft (SFB 1615 –503850735).

## Conflicts of Interest

The authors declare no conflicts of interest.

## Supporting information

Supplementary Material

## Data Availability

Research data is available after official publication (https://doi.org/10.15480/882.16323).
