# Supplementary material for: Investigations on the Role of Support and Synthesis Procedure for the Rux–Cuy/CNT‐Catalysed Hydrogenolysis of Glycerol to 1,2‐Propanediol
Source: ChemistryOpen. 2026 Jul 14;15(7):e70262. doi: 10.1002/open.70262 (PMC13370110; doi:10.1002/open.70262)
Supplement: Supplementary file 1 — Supplementary Material [file OPEN-15-e70262-s001.pdf]

Supporting Information for:

Investigations on the role of support and synthesis procedure for the Ru-Cu_X/CNT-catalysed hydrogenolysis of glycerol to 1,2-propanediol

The supporting information contains 32 figures and 8 tables on 33 pages

Experimental setup

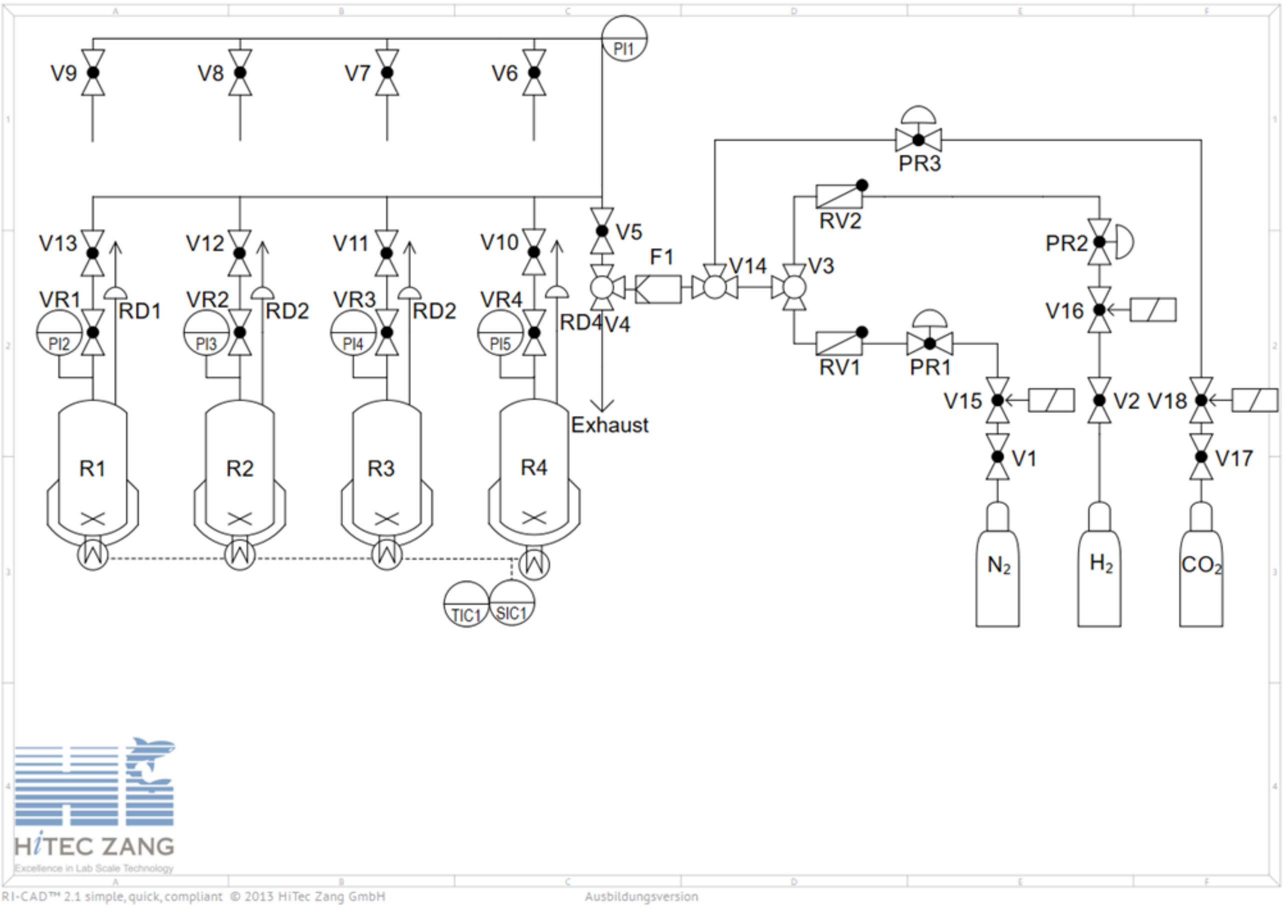

Figure S1: Flowsheet of the 4-fold multi batch reactor setup.

Elemental and textural analysis of the synthesized catalysts

Sample digestion for ICP-OES:

Before digestion, samples were usually freeze-dried first to eliminate any effect of varying water contents. For sample digestion, samples were digested via microwave-assisted acid digestion within sealed quartz vessels. To do so, usually between 5 and 10 mg (due to sample quantity limitations) of sample were put in the quartz vessels. Usually, samples were digested twice due to reasons of quality control (reproducibility). After that, 2 ml of concentrated suprapur HNO_3 were added. Samples were allowed to calm down if a strong chemical reaction appeared. Afterward, 1 ml of suprapur concentrated HCl and 1 ml of concentrated suprapur H_2SO_4 was added. The sealed quartz vessels were hermetically closed and therefore protected against potential losses of Ru (as RuO4) as soon as the sealing procedure (closing with PTFE strip and quartz lid and wrapping with PTFE strip) was finished. The samples were then placed within a Microwave 7000 (Anton Paar TM) for digestion. Following parameters were chosen for digestion: 25 min heating to 250 °C, 25 min keeping 250 °C, initial pressure of 60 bars, cooling temperature at 35 °C and a pressure decreasing rate of 2 bar/min. After digestion, sample solutions were usually clear, and the CNT material was dissolved completely. To ensure that Ru is not lost as its volatile species, RuO_4 (boiling point of 40 °C), samples cooled down to 4°C before opening again. Samples were then filtered and diluted to final acid concentration for analyses via ICP-OES. For better yields, the individual quartz vessels were washed with 5 ml of 1 M HCl during filtration process.

Table S1: ICP-OES results of the synthesized catalysts.

**Catalyst**	**Theoretical wt% / g/kg**		**ICP-OES wt% / g/kg**		**ICP-OES loading / %**	**Ratio Cu/Ru**
Ru-Cu_3/NC7000[1]	Ru-17.4	Cu-33.1	Ru-12.1	Cu-37.8	4.99	
Ru-Cu_3/Baytubes	Ru-17.4	Cu-32.6	Ru-10.5	Cu-17.3	2.78	1.65
Ru-Cu_3/Flycarbon	Ru-18.1	Cu-31.9	Ru-15.6	Cu-28.7	4.43	
Ru-Cu_3/TuBalls	Ru-17.4	Cu-32.7	Ru-15.3	Cu- < 5	< 2.03	
Ru-Cu_3/Globugraphite	Ru-17.9	Cu-33.8	Ru-14.8	Cu-19.4	3.42	
Ru-Cu_5/Baytubes	Ru-12.2	Cu-37.9	Ru-7.8	Cu-25.7	3.35	3.28
Ru-Cu_4/Baytubes	Ru-14.2	Cu-36.0	Ru-9.7	Cu-27.5	3.72	2.82
Ru-Cu_2/Baytubes	Ru-22.2	Cu-27.9	Ru-22.0	Cu-26.7	4.95	1.21
Ru-Cu/Baytubes	Ru-30.7	Cu-19.3	Ru-20.1	Cu-14.5	3.46	0.72
Ru_2-Cu/Baytubes	Ru-38.0	Cu-11.8	Ru-31.9	Cu-9.8	4.17	0.31
Ru_3-Cu/Baytubes	Ru-41.4	Cu-8.7	Ru-31.9	Cu-4.89	3.62	0.15
Ru-Cu_3/Baytubes (N)	Ru-17.6	Cu-32.8	Ru-15.5	Cu-23.9	3.94	
Ru-Cu_3/Baytubes (Cl)	Ru-17.5	Cu-32.7	Ru-10.9	Cu-18.9	2.98	
Ru-Cu_3/Baytubes (OAc)	Ru-17.6	Cu-32.6	Ru-13.6	Cu-19.4	3.30	
Ru-Cu_3/Baytubes (acac)	Ru-17.5	Cu-32.8	Ru-10.7	Cu-15.8	2.65	
Ru-Cu_3/Baytubes-IWI-H_2O	Ru-17.3	Cu-32.6	Ru-12.8	Cu-24.3	3.71	
Ru-Cu_3/Baytubes-IWI-EtOH	Ru-17.3	Cu-32.7	Ru-13.5	Cu-26.8	4.03	
Ru-Cu_3/Baytubes-CD	Ru-17.4	Cu-32.7	Ru-15.8	Cu-33.1	4.89	
Ru-Cu_3/Baytubes-DP	Ru-17.3	Cu-32.8	Ru-12.5	Cu-25.3	3.78	
Ru-Cu_3/Baytubes-CP	Ru-17.3	Cu-33.0	Ru-15.1	Cu-27.9	4.30	
Ru-Cu_3/Baytubes-SSG	Ru-17.6	Cu-32.9	Ru-15.3	Cu-27.4	4.27	
Ru-Cu_3/Baytubes-BMSSG	Ru-17.3	Cu-32.7	Ru-6.75	Cu-6.73	1.35	
Ru-Cu_3/Baytubes after the recycling study			Ru-9.9	Cu < 5.0	< 14.9	

Table S2: Point of zero charge of the investigated supports.

Support	Point of zero charge
**NC7000**	6.72
**Baytubes**	7.88
**Flycarbon**	7.17
**TuBalls**	7.38
**Globugraphite**	6.67

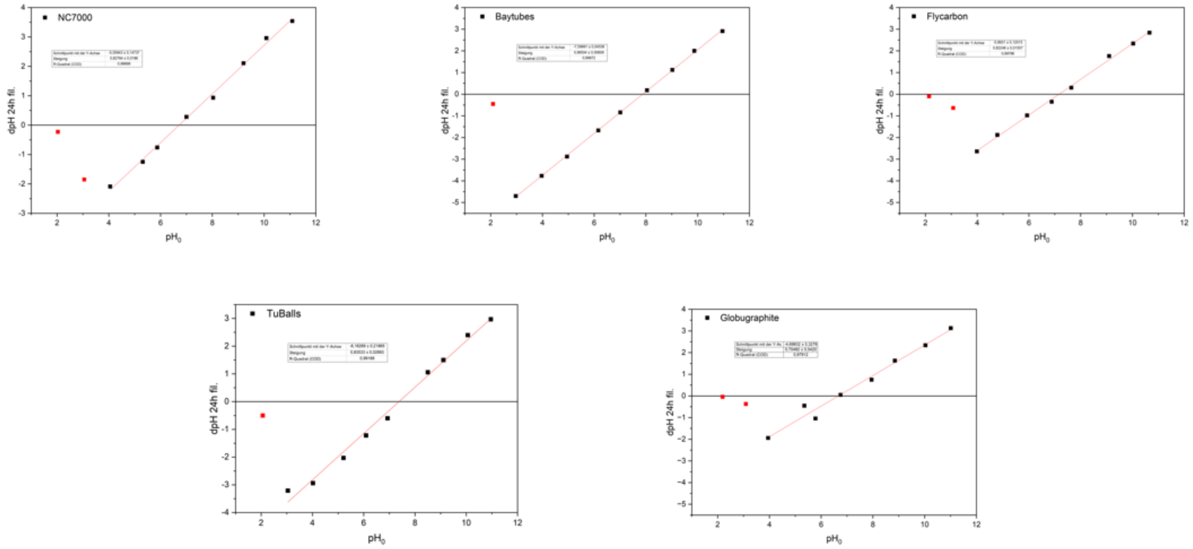

Figure S2: Point of zero charge plots for the different supports.

<u>Morphological investigations of the synthesized catalysts</u>

The applicability of the applied textural models was verified prior to discussion of the structural trends. The BET evaluation was performed within the relative pressure range p/p_0 = 0.025–0.31 and fulfilled the Rouquerol consistency criteria for all samples. The corresponding BET plots exhibited high linearity and the calculated C constants were positive for all materials except TuBalls, confirming adequate adsorbate–adsorbent interaction and model validity in the majority of cases (Figures S9–S10, Table S3).

To further ensure model independence, pore structure was additionally evaluated using QSDFT (equilibrium model, cylindrical pore assumption). Although absolute values differ slightly from BET/BJH results, identical trends are observed (Table S3, Figure S11 and S12). Overall, BJH- and QSDFT-derived mesopore volumes show consistent trends with only minor deviations. The larger discrepancies observed for $RuCu_3$/TuBalls and $RuCu_3$/Globugraphite (with BJH yielding higher V_{meso}) likely arise from hysteresis-related network effects (pore blocking/ink-bottle behaviour) that may lead to higher apparent mesopore volumes in BJH analysis of the desorption branch, whereas QSDFT provides a more conservative estimate for irregular and hierarchical pore architectures. t-plot analysis was performed to assess potential microporosity contributions (Figure S13 and S14). Except for TuBalls, no measurable

micropore surface area or micropore volume was detected (Table, S3), confirming that the investigated materials are predominantly mesoporous.

Since the added metal mass cannot explain these changes, structural effects arising during wet impregnation and ambient drying must be considered. Introducing an aqueous precursor solution exposes the supports to capillary forces that may contract CNT bundles within rigid MWCNT aggregates, thereby reducing pore accessibility. Such capillary-force-induced contraction or partial mesopore narrowing represents a plausible contributing factor, complementing pore blockage by deposited metal species.

Conversely, in structurally more flexible or hierarchically organized materials such as TuBalls and Globugraphite, impregnation and subsequent thermal treatment can enhance pore accessibility. Removal of adsorbed species, partial debundling of SWCNT networks, or improved connectivity between pre-existing cavities may increase both the accessible surface area and the apparent mesopore volume. Indeed, both TuBalls and Globugraphite exhibit a pronounced increase in surface area and mesopore volume after metal loading. Notably, $RuCu_3$@TuBalls is the only material exhibiting a pronounced increase in micropore surface area according to t-plot analysis. This suggests that impregnation and subsequent thermal treatment not only enhance mesopore accessibility but also render narrow slit-like interbundle voids accessible to N_2. Such microporosity is consistent with the structural characteristics of SWCNT bundle networks, where interstitial spaces may initially be partially inaccessible. The absence of comparable micropore contributions in the other materials indicates that this effect is specific to the SWCNT-based support and not a general consequence of metal deposition.

These observations suggest that wet impregnation induces support-dependent textural reorganization rather than a uniform pore-blocking effect. The increase in mesopore volume observed for TuBalls and Globugraphite indicates a measurable restructuring of the carbon framework or opening/widening of pre-existing voids, whereas in Flycarbon partial contraction or limited accessibility dominates.

Although these interpretations cannot be directly verified by the available structural data, they are consistent with the combined BET, BJH, QSDFT and t-plot analyses.

Together, these observations demonstrate that the evolution of pore structure is governed by support-specific structural responses to impregnation and reduction rather than by the metal mass itself. Supports with inherently broad and accessible mesopore networks, such as Baytubes, retain favourable mass transport properties after loading, whereas materials with limited initial porosity, such as Flycarbon, lose accessibility. More structurally adaptable systems, including TuBalls and Globugraphite, can develop substantially more open pore networks upon metal incorporation. These differences in textural evolution correlate closely with the catalytic behaviour of the Ru–Cu catalysts and highlight the decisive role of support architecture in determining accessibility and dispersion of the active phase. In addition to pore structure, surface chemical properties may also influence metal deposition and particle growth. The point of zero charge of the supports lies between 6.67 and 7.88 (Figure S2), indicating comparable surface charge characteristics under the applied preparation conditions.

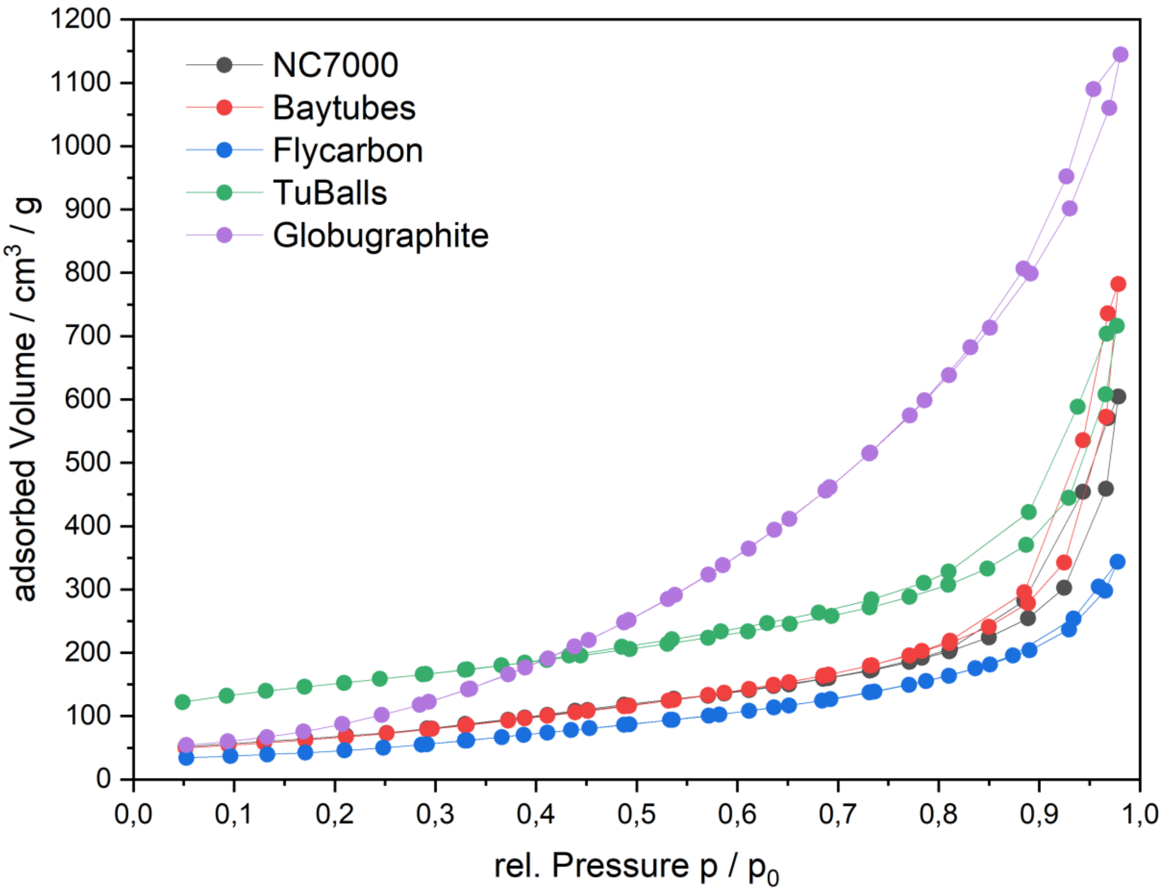

Figure S3: N_2-physisorption isotherms of the used carbon materials.

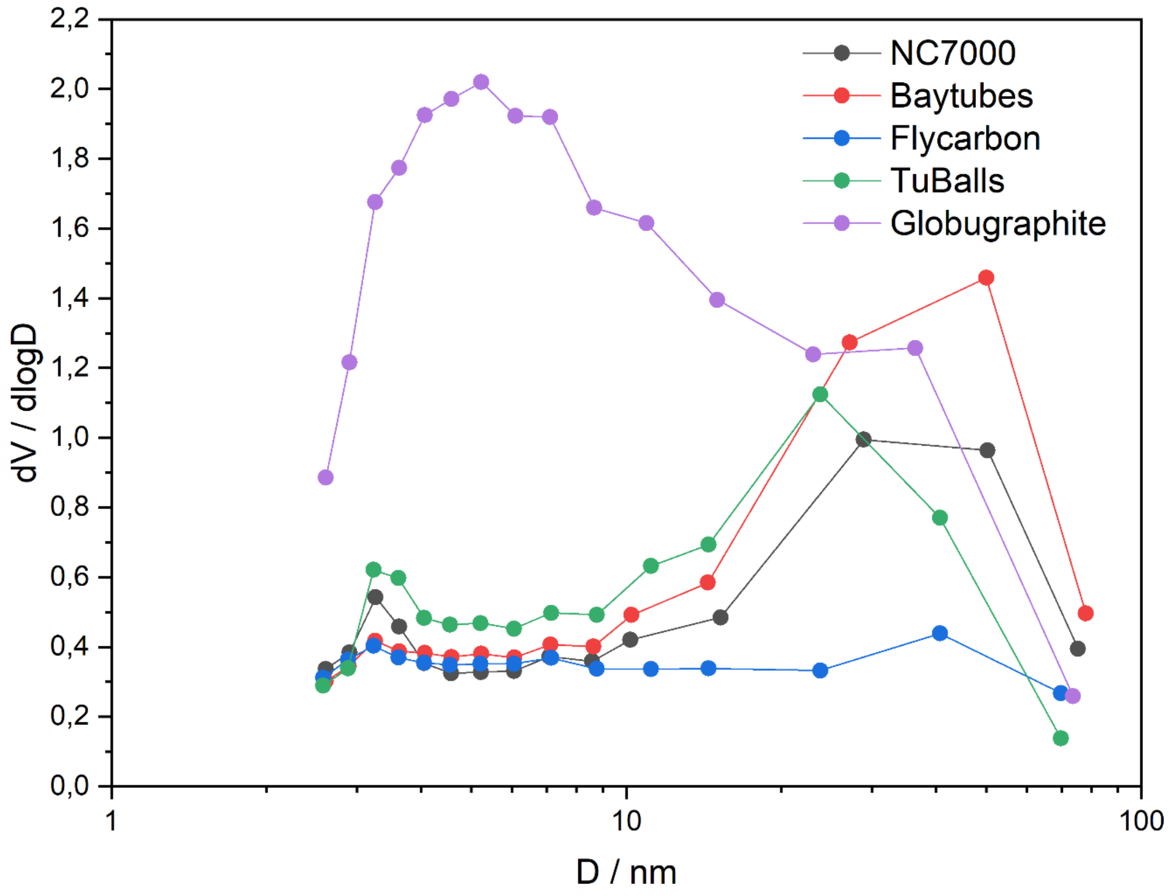

Figure S4: Pore size distribution of the used carbon materials.

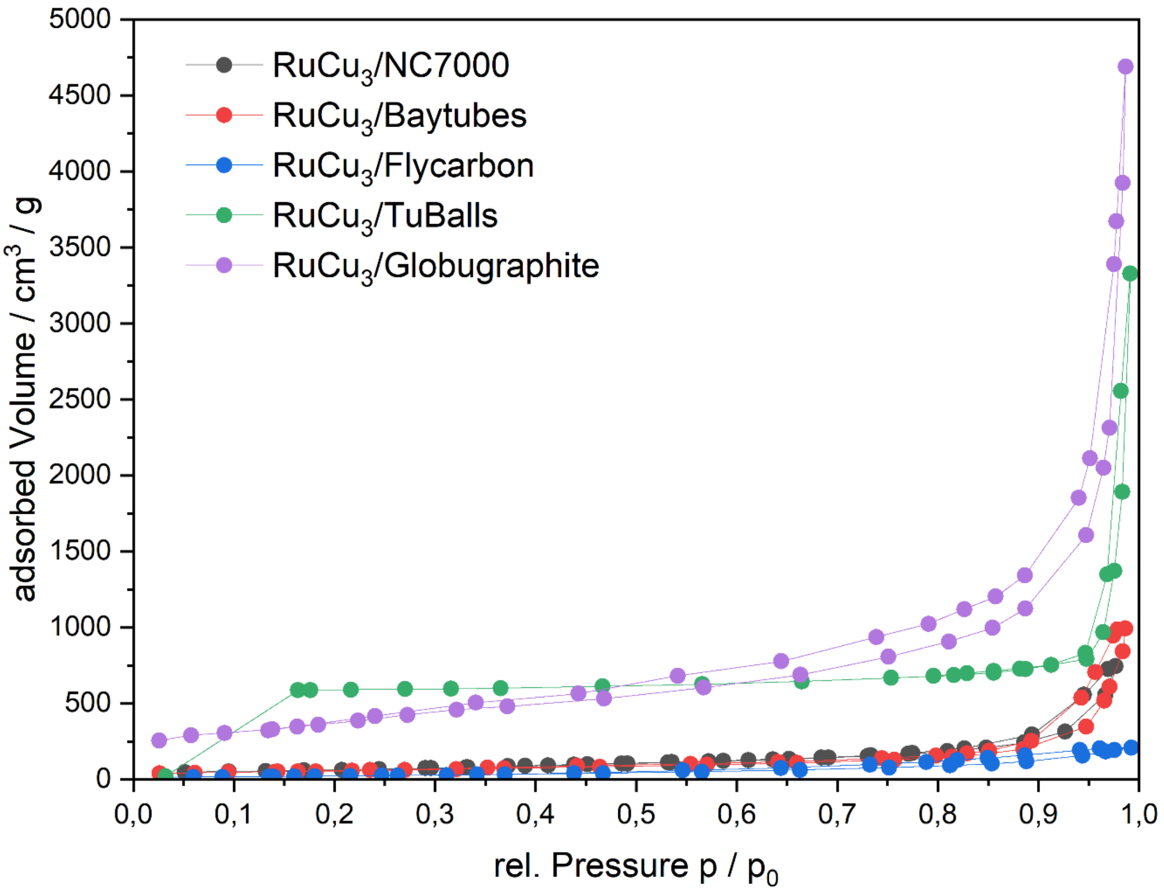

Figure S5: N_2-physisorption isotherms of the used carbon supported catalysts.

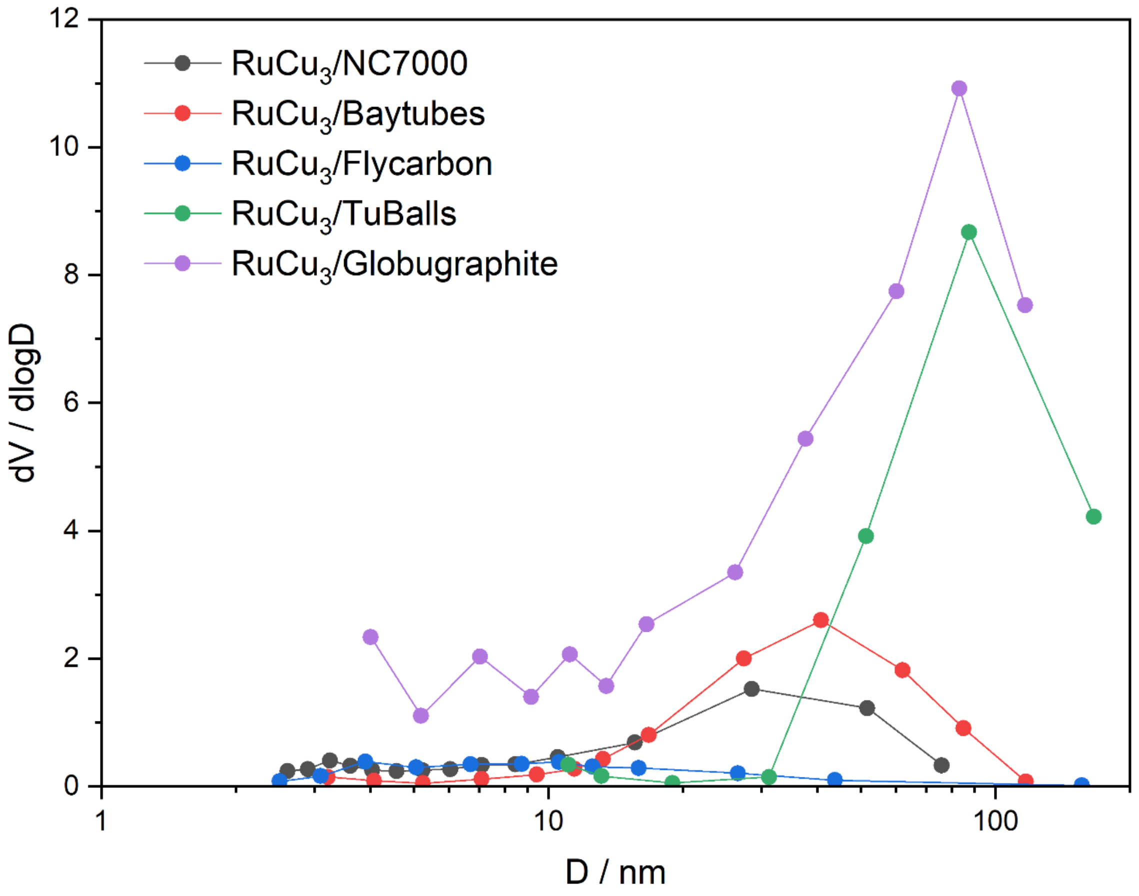

Figure S6: Pore size distribution of the used carbon supported catalysts.

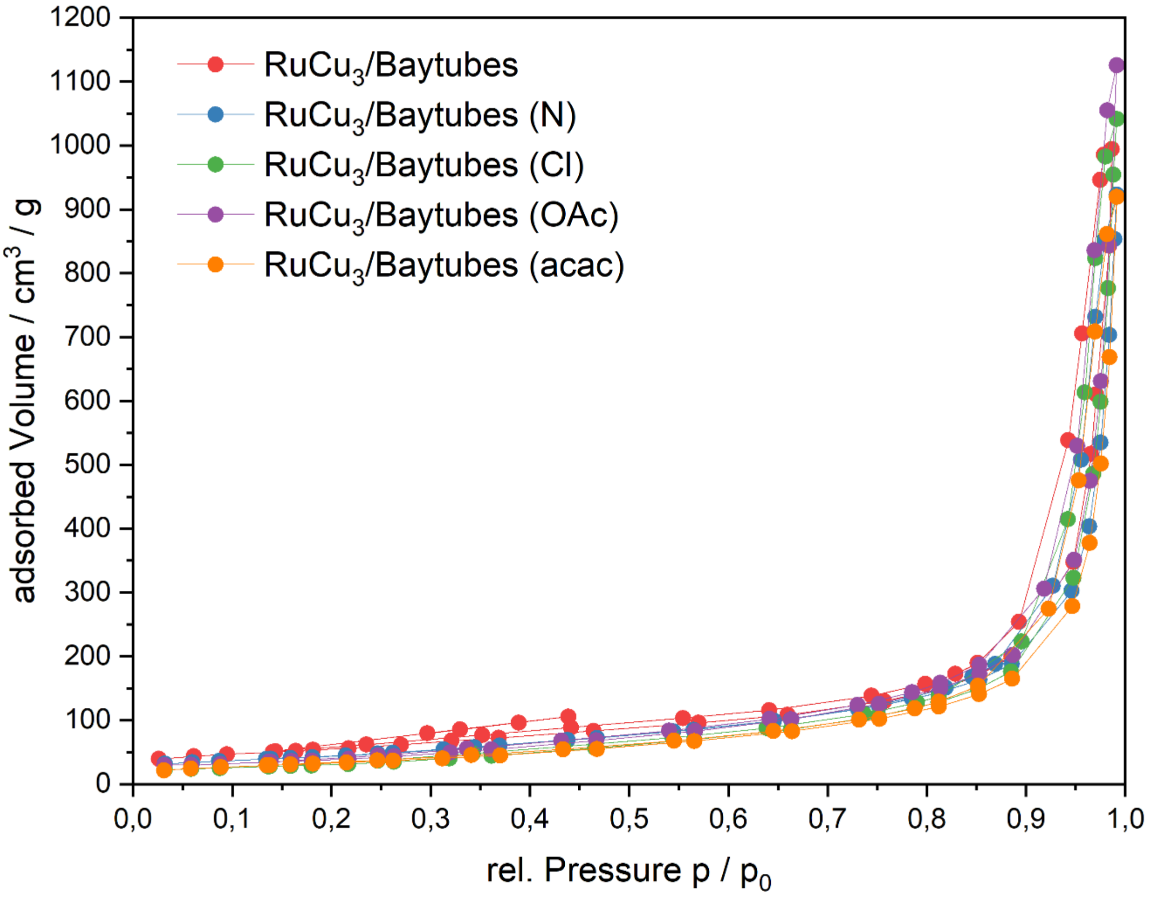

Figure S7: N_2-physisorption isotherms of the catalysts were different Ru and Cu precursors were used during the synthesis.

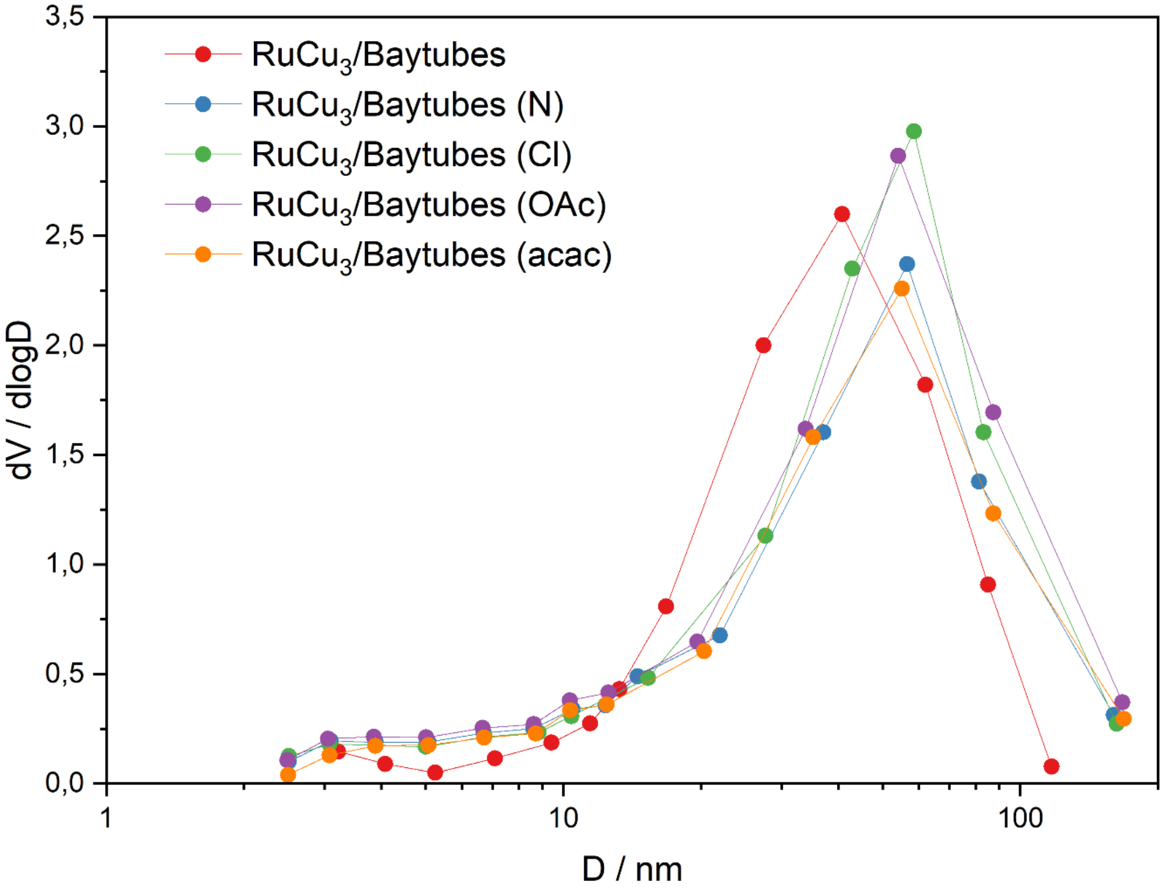

Figure S8: Pore size distribution of the catalysts used in the precursor variation.

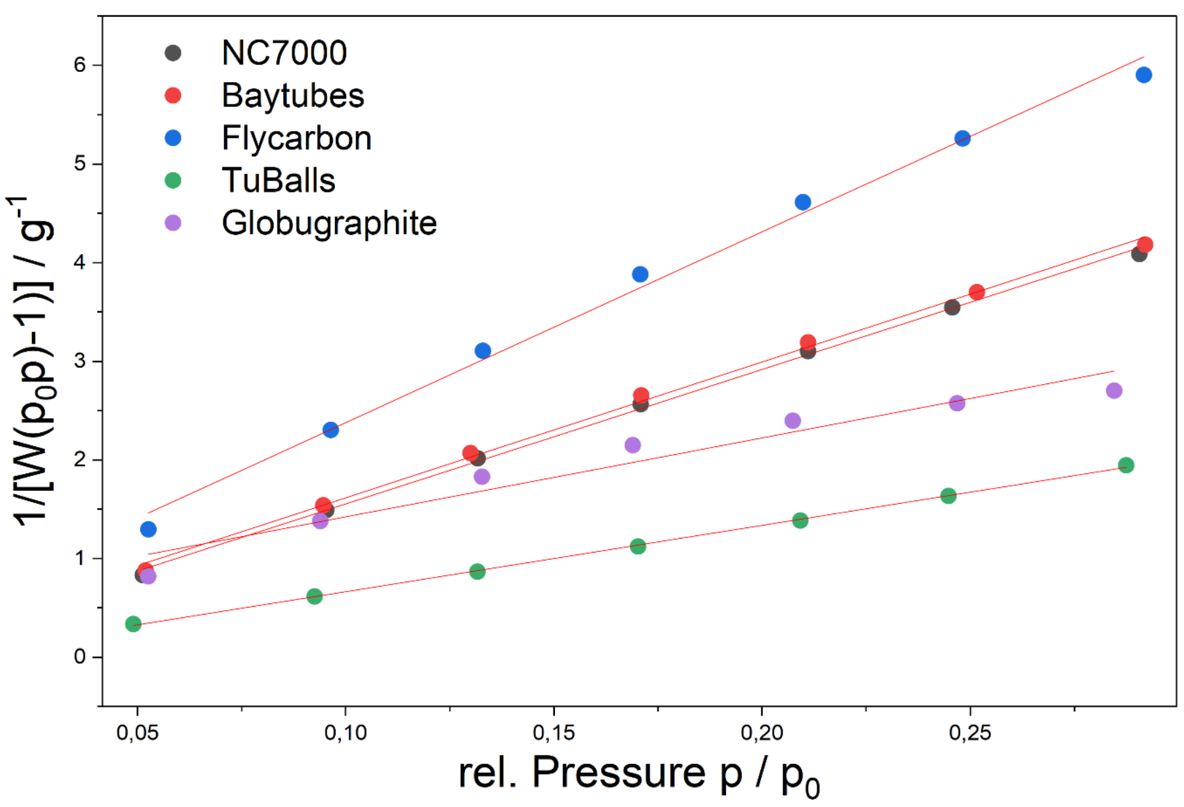

Figure S9: Plots of the BET evaluation in the range of p/p_0 = 0.025–0.31 for the support materials.

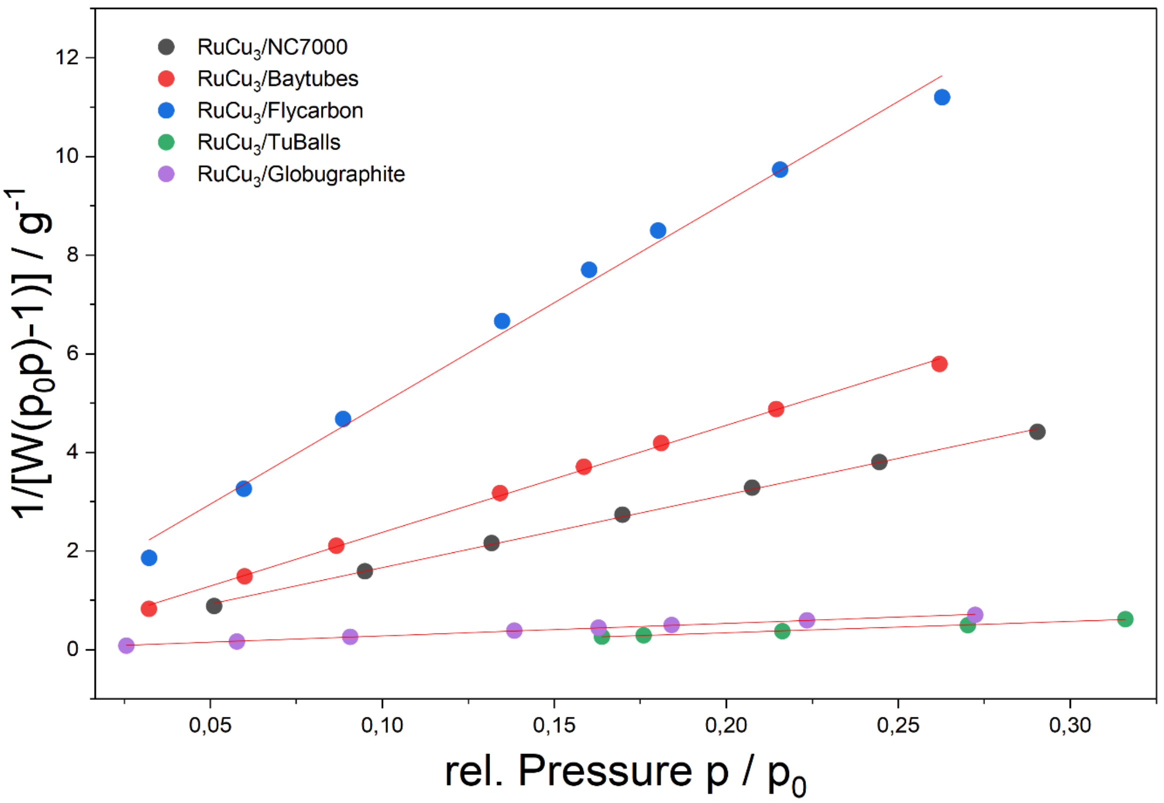

Figure S10: Plots of the BET evaluation in the range of p/p_0 = 0.025–0.31 for the different carbon supported catalysts.

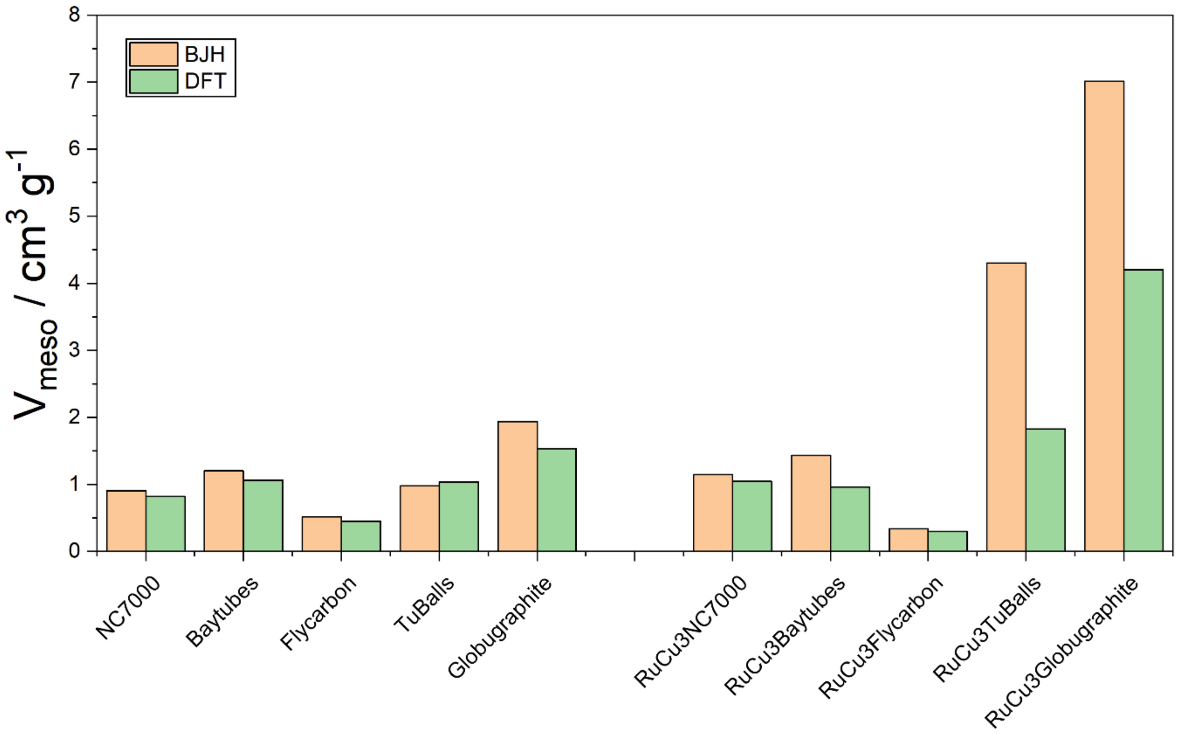

Figure S11: Comparison between the BJH- and QSDFT-derived mesopore volumes.

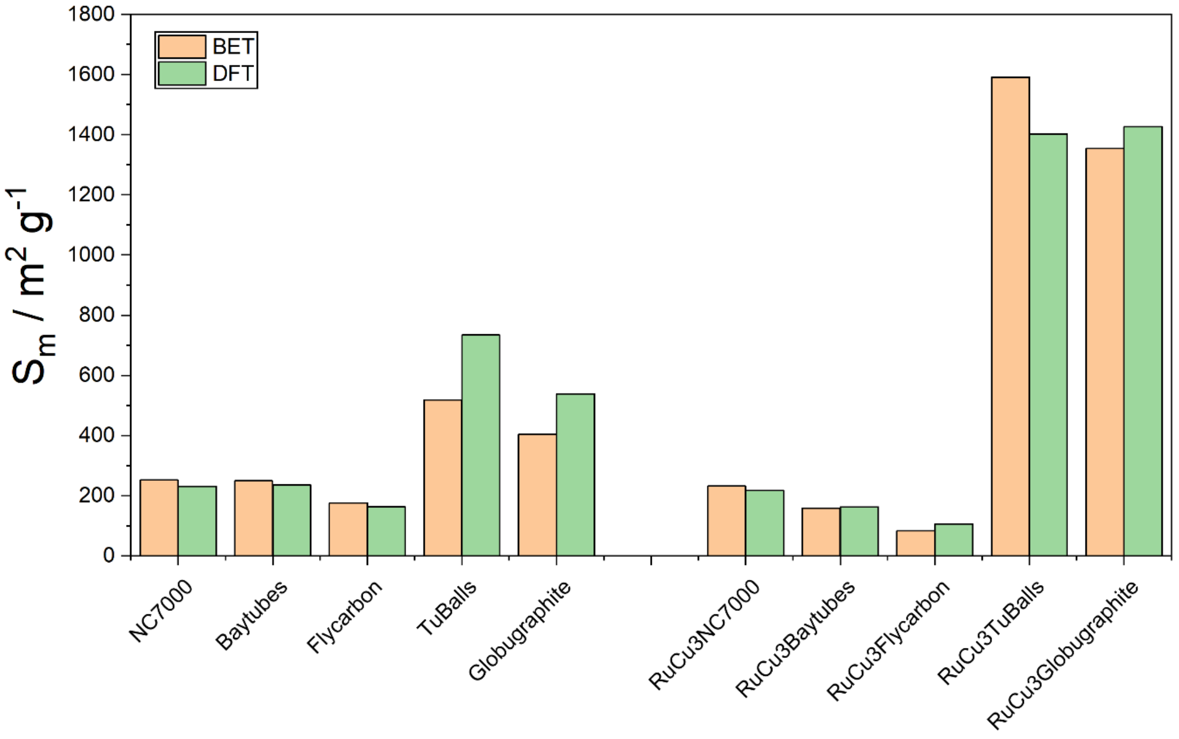

Figure S12: Comparison between the BET- and QSDFT-derived surface areas.

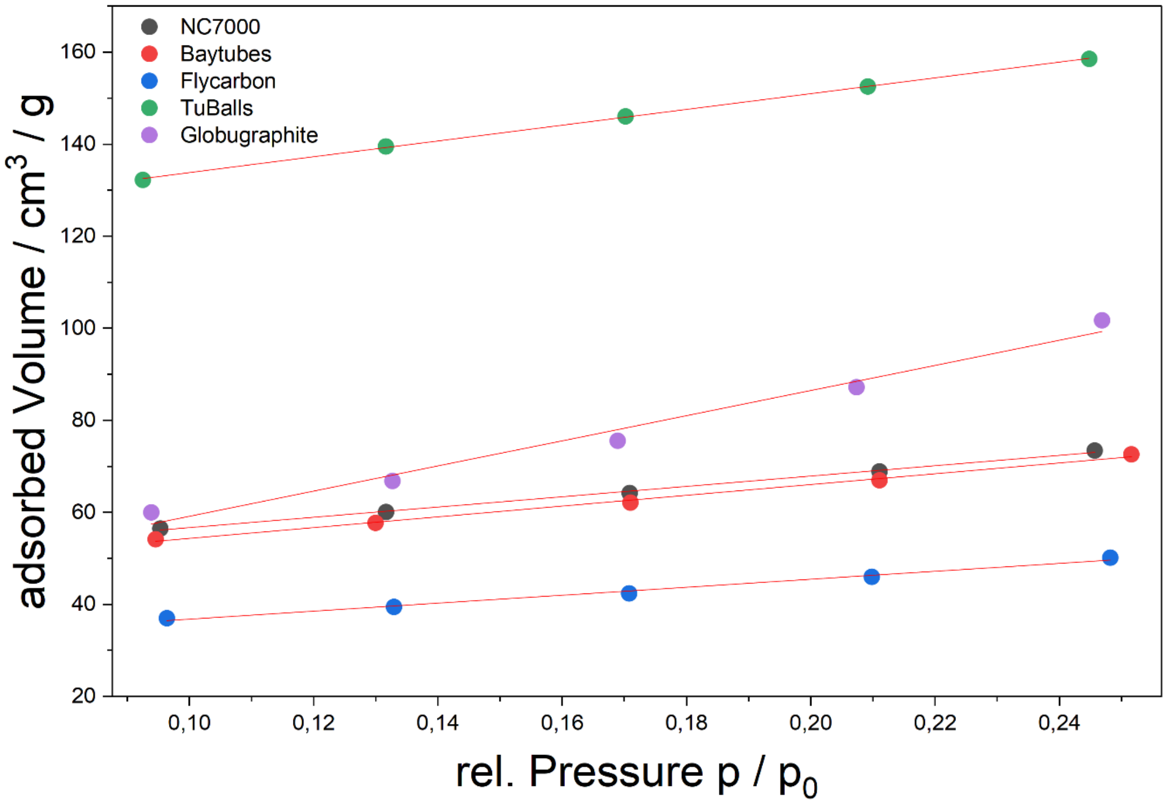

Figure S13: t-plot analysis of the support materials.

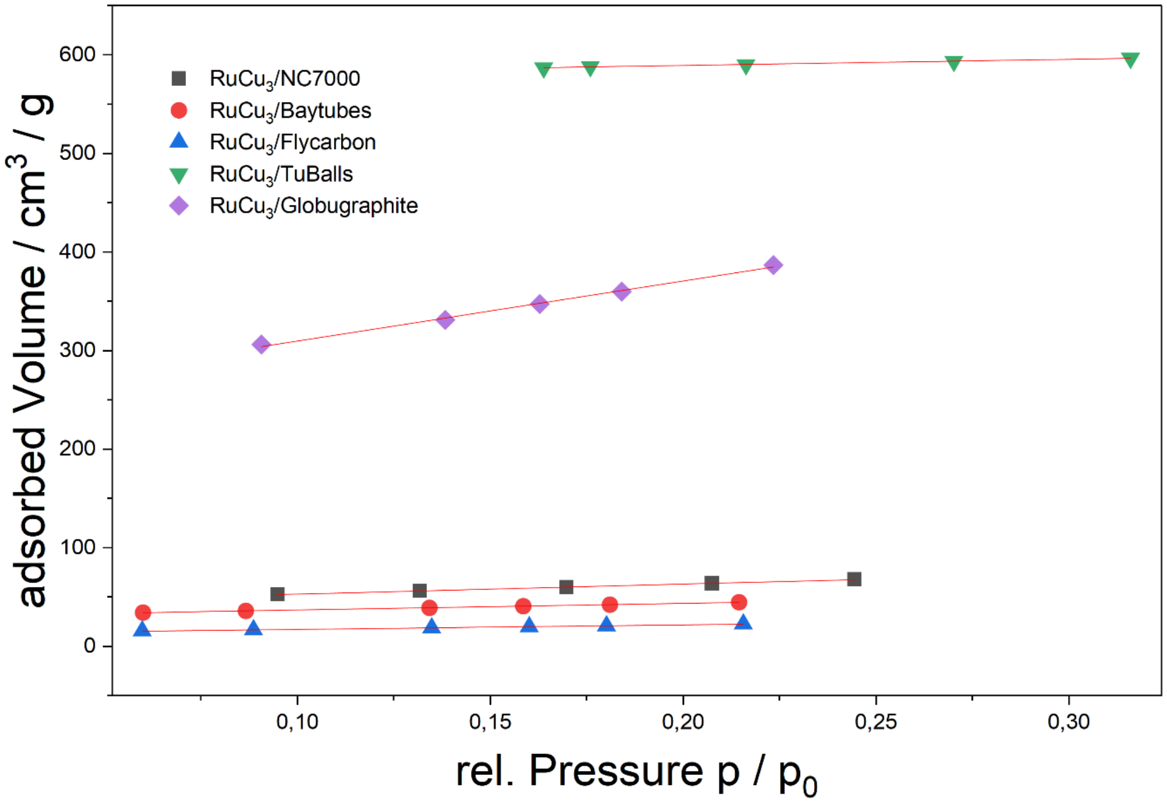

Figure S14: t-plot analysis of the catalysts on the different support materials.

Table S3: Additional data for BET, BJH and DFT analysis as well as the t-plots.

Catalyst	S_m BET	R^2 BET	C BET	V_{meso}	S_m DFT	V_{meso} DFT	S_m Extern	S_m micro	V_{micro}	R^2 t-Plot
**NC7000**	252	0,9993	72	0,906	231	0,818	252	0	0	0,9968
**Baytubes**	249	0,9989	58,2	1,202	235,3	1,063	249	0	0	0,9957
**Flycarbon**	176	0,9969	44,4	0,518	163	0,451	176	0	0	0,9926
**TuBalls**	518	0,9997	-852	0,977	734,2	1,036	391,3	126,9	0,063	0,9999
**Globugraphite**	404	0,9719	14	1,94	537,8	1,534	404	0	0	0,9857
**Ru-Cu_3/NC7000**	232	0,9994	80	1,145	217,4	1,045	232	0	0	0,9968
**Ru-Cu_3/Baytubes**	159	0,9993	105	1,43	162,3	0,96	143,2	15,6	0,005	0,9963
**Ru-Cu_3/Flycarbon**	83,4	0,9963	46	0,34	105,4	0,297	83,4	0	0	0,9944
**Ru-Cu_3/TuBalls**	1591	0,9988	18,8	4,301	1402	1,826	149,388	1442	0,846	0,9987
**Ru-Cu_3/Globugraphite**	1354	0,9992	98,1	7,011	1427	4,201	1354	0	0	0,9955

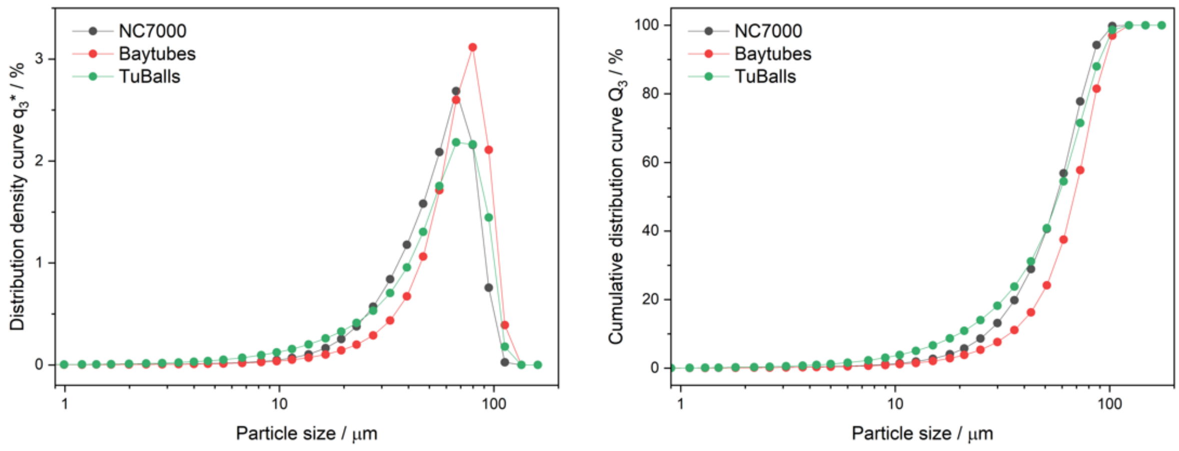

Figure S15: Particle distribution density curve and cumulative distribution for selected supports.

Table S4: Physico-chemical properties of additional metal impregnated catalysts. **a:** Determined with N_2-physisorption using the BET method **b:** Determined with N_2-physisorption using the BJH method.

**Catalyst**	**[a]Total surface area (m^2g^{-1})**	**[b]Pore volume (cc/g)**	**[b]Pore diameter (nm)**
Ru-Cu_5/Baytubes	171.5		
Ru-Cu_4/Baytubes	183.2		
Ru-Cu_2/Baytubes	153.0		
Ru-Cu/Baytubes	168.3		
Ru_2-Cu/Baytubes	150.9		
Ru_3-Cu/Baytubes	179.3		
Ru-Cu_3/Baytubes (N)	160.6	1.435	35.5
Ru-Cu_3/Baytubes (Cl)	114.0	1.639	56.5
Ru-Cu_3/Baytubes (OAc)	142.4	1.762	48.9
Ru-Cu_3/Baytubes (acac)	122.6	1.435	46.4
Ru-Cu_3/Baytubes-IWI-H_2O	179.7		
Ru-Cu_3/Baytubes-IWI-EtOH	169.3		
Ru-Cu_3/Baytubes-CD	223.3		
Ru-Cu_3/Baytubes-DP	229.4		
Ru-Cu_3/Baytubes-CP	234.6		
Ru-Cu_3/Baytubes-SSG	167.0		
Ru-Cu_3/Baytubes-BMSSG	225.0		
Ru-Cu_3/Baytubes after the recycling study	203.3	1.316	17.0

Table S5: Average particle sizes for some selected supports.

**Support material**	**X_{50} (µm)**
NC7000	56.79
Baytubes	68.41
TuBalls	57.71

Microscopy

RuCu$_3$/Baytubes (N) RuCu$_3$/Baytubes (Cl) RuCu$_3$/Baytubes (OAc) RuCu$_3$/Baytubes (acac)

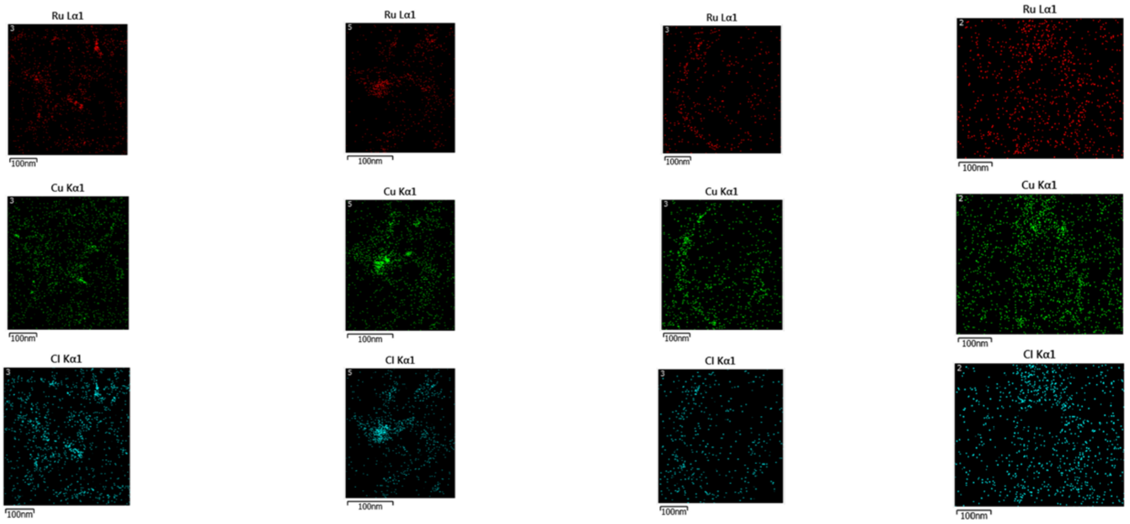

Figure S16: TEM-EDX mapping of the Ru-Cu₃/Baytubes catalyst before the reduction, were different metal precursors were used.

NC7000 Baytubes Flycarbon TuBalls Globugraphite

RuCu$_3$/Baytubes (N) RuCu$_3$/Baytubes (Cl) RuCu$_3$/Baytubes (OAc) RuCu$_3$/Baytubes (acac)

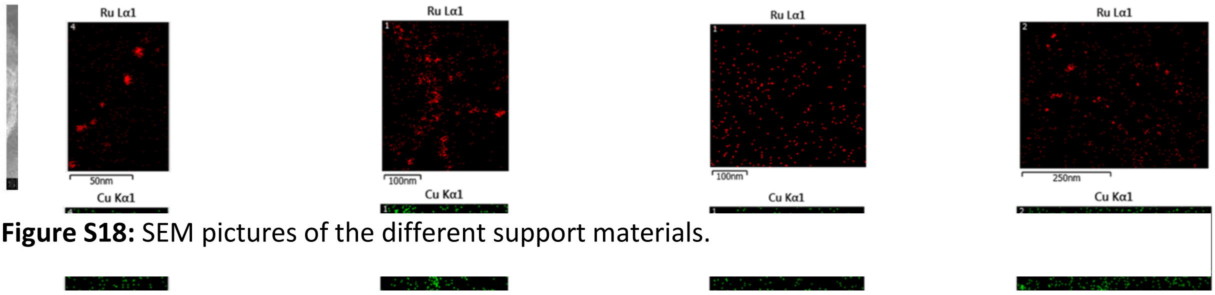

Figure S18: SEM pictures of the different support materials.

NC7000 Baytubes Flycarbon TuBalls Globugraphite

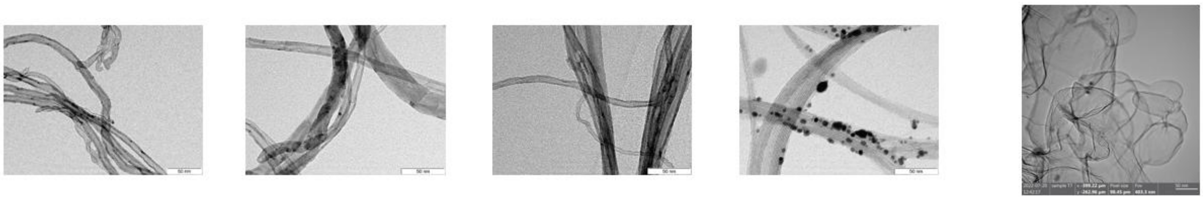

Figure S19: TEM pictures of the different support materials.

metal precursors were used.

RuCu$_3$/NC7000 RuCu$_3$/Baytubes RuCu$_3$/Flycarbon RuCu$_3$/TuBalls

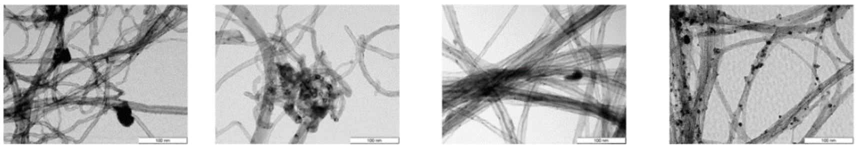

Figure S20: TEM pictures of the Ru-Cu catalysts supported on different supports.

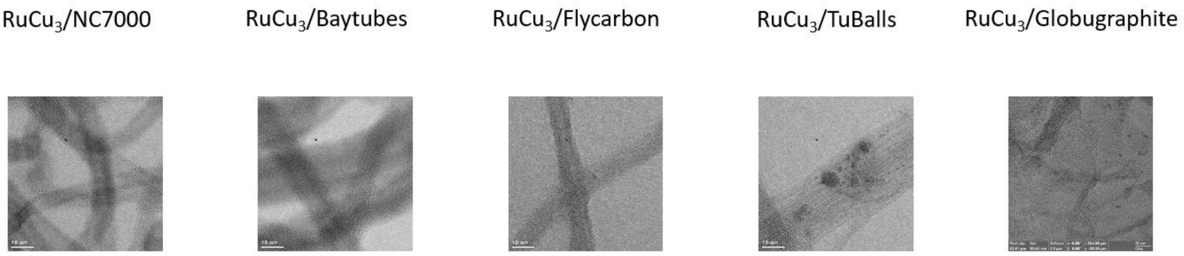

Figure S21: HR-TEM pictures of the Ru-Cu catalysts supported on different supports.

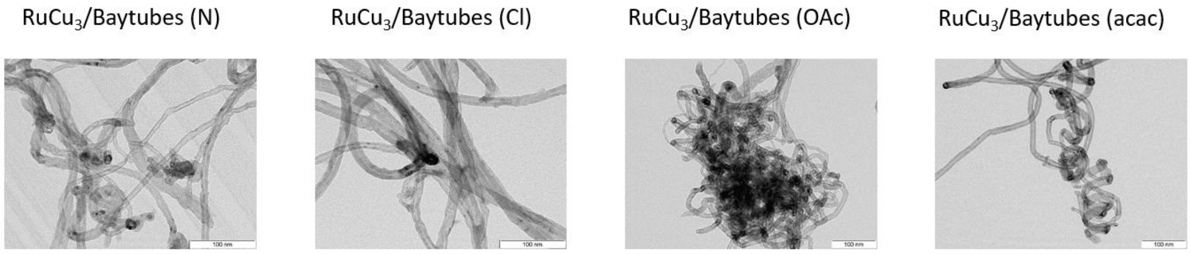

Figure S22: TEM pictures of the catalysts used in the precursor variation.

Powder-X-ray structural investigations of the synthesized catalysts

Figure S*23*: Diffractograms oft the support materials.

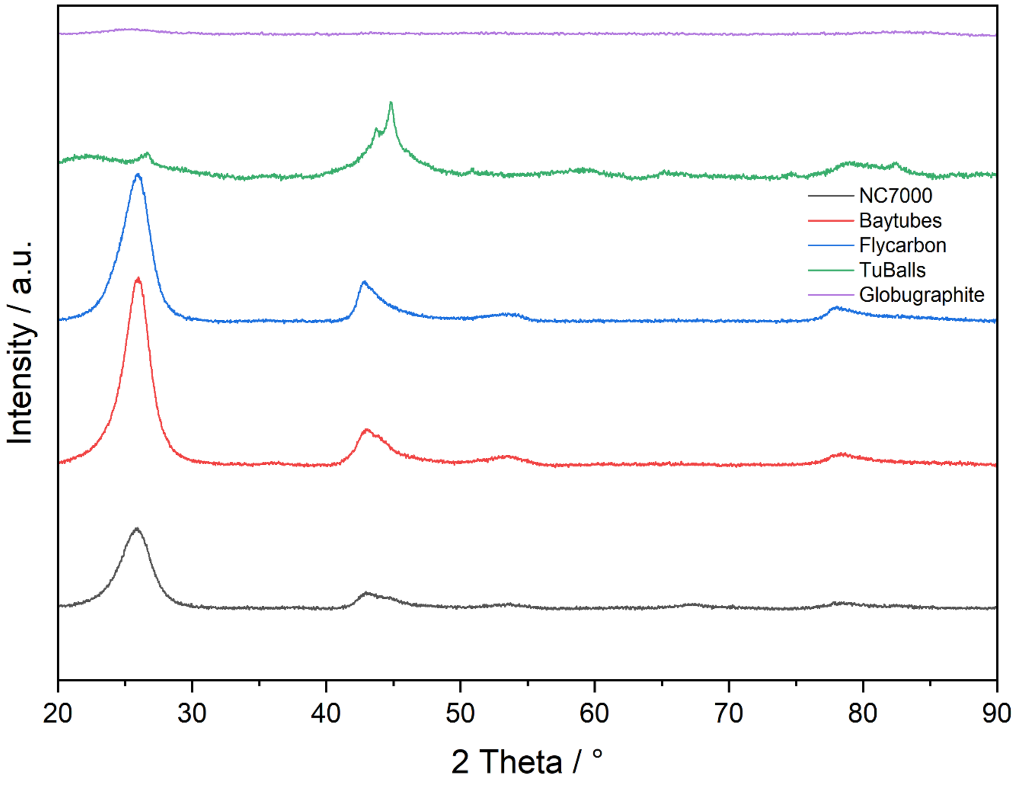

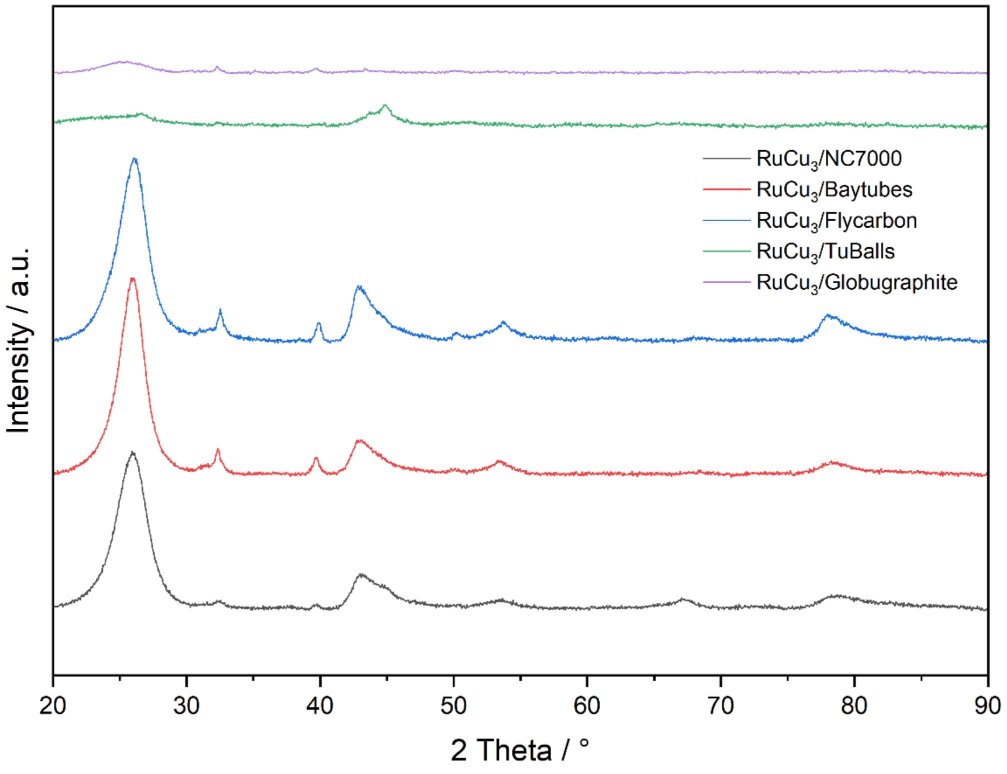

Figure S24: Diffractograms oft the supported catalysts.

Additional experimental data

Table S6: Retention times of the detectable liquid substances.

Retention time / min	Substance
9.96	Glycerol
12.01	Ethylene glycol
12.44	1,2 Propanediol
12.92	1,3 Propanediol
13.40	Acetol
14.34	Methanol
15.78	Ethanol
16.80	iso-Propanol
19.85	n-Propanol

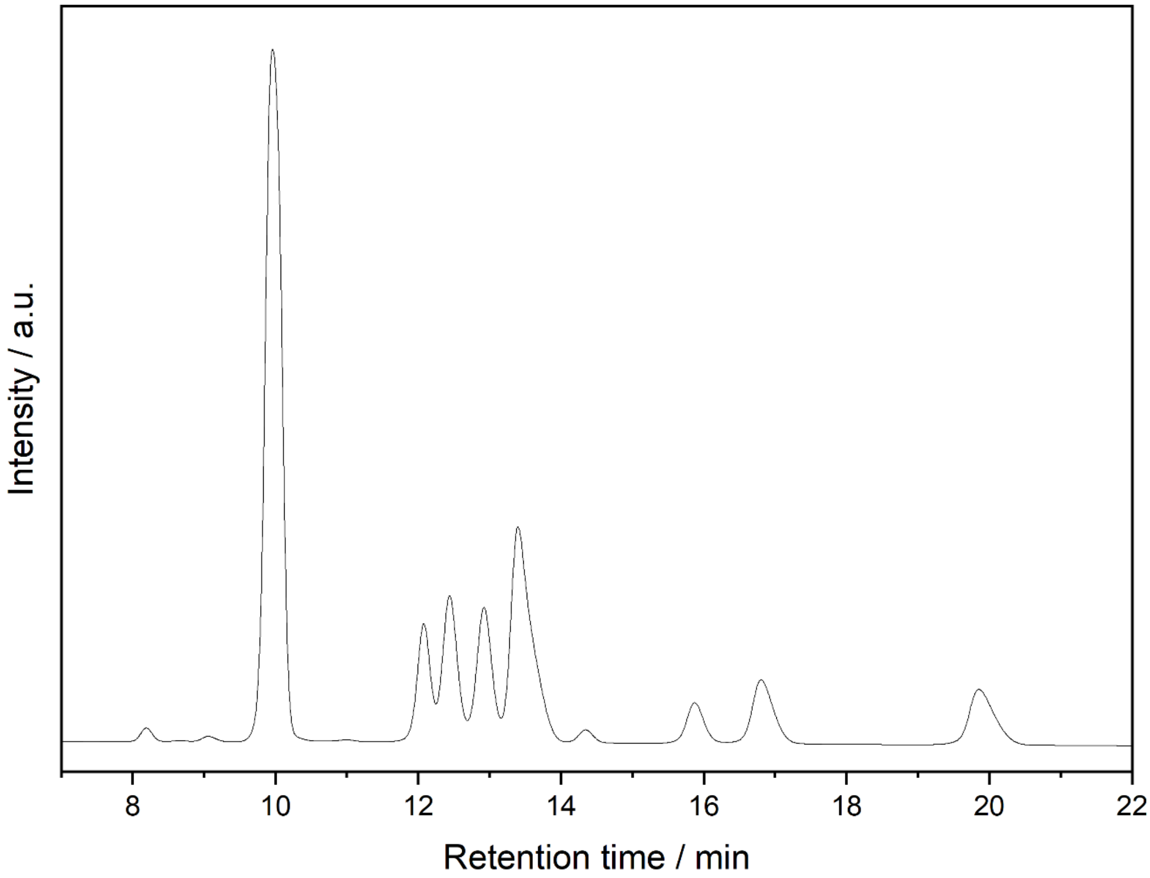

Figure S25: HPLC Chromatogram of a test mixture containing all detectable liquid substances.

==== Shimadzu LabSolutions Calibration Curve ====

ID# : 1
Name : Methanol
Quantitative Method : External Standard
Function : f(x)=472009*x+1693.73
Rr1=0.9999813 Rr2=0.9999625 RSS=1.154804e+008
MeanRF: 5.053024e+005 RFSD: 5.130887e+004 RFRSD: 10.154092
FitType : Linear
ZeroThrough : Not Through
Weighted Regression : None
Offset Correction : Off
Detector Name : Detector A

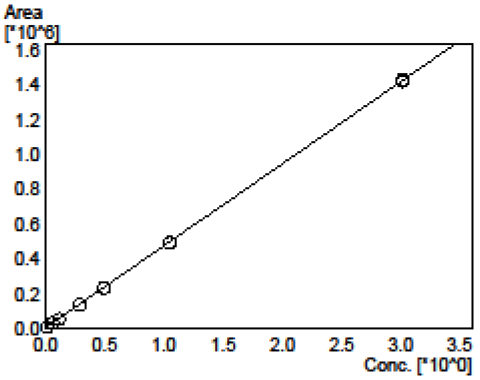

#	Conc.(Ratio)	MeanArea	Area
1	3.016738	1421611	1421611
2	1.049395	491568	491568
3	0.4943277	233649	233649
4	0.2893701	137209	137209
5	0.1127578	54855	54855
6	0.06136267	33458	33458
7	0.01619725	9961	9961
8	3.016738	1432378	1432378
9	1.049395	494991	494991
10	0.4943277	235437	235437
11	0.2893701	138098	138098
12	0.1127578	55153	55153
13	0.06136267	33521	33521
14	0.01619725	9809	9809

ID# : 2
Name : Glycerin
Quantitative Method : External Standard
Function : f(x)=7.32678e+006*x+257390
Rr1=0.9990066 Rr2=0.9980143 RSS=1.420324e+012
MeanRF: 7.967319e+006 RFSD: 3.432594e+005 RFRSD: 4.308342
FitType : Linear
ZeroThrough : Not Through
Weighted Regression : None
Offset Correction : Off
Detector Name : Detector A

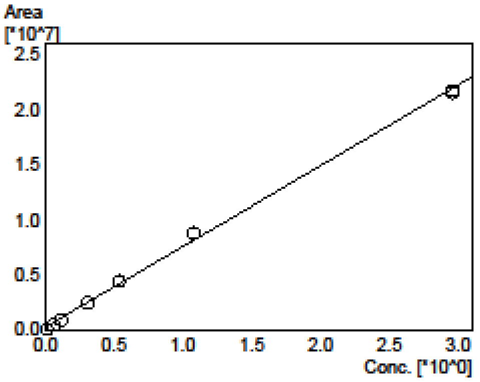

#	Conc.(Ratio)	MeanArea	Area
1	2.958375	21755658	21755658
2	1.078646	8829660	8829660
3	0.5353454	4460290	4460290
4	0.3066948	2502696	2502696
5	0.114594	912734	912734
6	0.05901699	480348	480348
7	0.0117174	89082	89082
8	2.958375	21567307	21567307
9	1.078646	8777924	8777924
10	0.5353454	4438634	4438634
11	0.3066948	2507066	2507066
12	0.114594	919111	919111
13	0.05901699	484280	484280
14	0.0117174	90038	90038

ID# : 3
Name : n-Propanol
Quantitative Method : External Standard
Function : f(x)=3.95575e+006*x-25854.6
Rr1=0.9999829 Rr2=0.9999658 RSS=7.510615e+009
MeanRF: 3.822135e+006 RFSD: 1.142245e+005 RFRSD: 2.988500
FitType : Linear
ZeroThrough : Not Through
Weighted Regression : None
Offset Correction : Off
Detector Name : Detector A

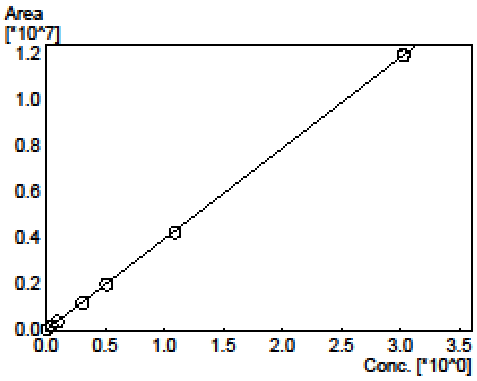

#	Conc.(Ratio)	MeanArea	Area
1	3.02526	11938714	11938714
2	1.092217	4248851	4248851
3	0.5139845	1989274	1989274
4	0.3105323	1181496	1181496
5	0.09785473	371245	371245
6	0.04497364	171409	171409
7	0.01087275	38786	38786
8	3.02526	11978515	11978515
9	1.092217	4264286	4264286
10	0.5139845	1996879	1996879
11	0.3105323	1187834	1187834
12	0.09785473	373727	373727
13	0.04497364	172565	172565
14	0.01087275	39096	39096

ID# : 4
Name : 1,2 Propandiol
Quantitative Method : External Standard
Function : f(x)=5.77107e+006*x+60077.2
Rr1=0.9998942 Rr2=0.9997884 RSS=9.499839e+010
MeanRF: 5.939932e+006 RFSD: 9.021970e+004 RFRSD: 1.518867
FitType : Linear
ZeroThrough : Not Through
Weighted Regression : None
Offset Correction : Off
Detector Name : Detector A

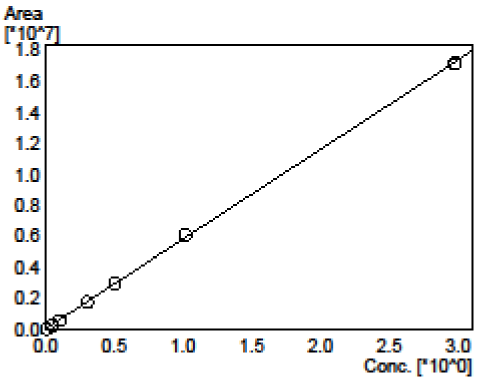

#	Conc.(Ratio)	MeanArea	Area
1	2.977546	17218092	17218092
2	1.016412	6105149	6105149
3	0.5030017	3019381	3019381
4	0.3027638	1798243	1798243
5	0.1023958	606721	606721
6	0.04970167	291995	291995
7	0.01157971	70080	70080
8	2.977546	17138745	17138745
9	1.016412	6097247	6097247
10	0.5030017	3010906	3010906
11	0.3027638	1801894	1801894
12	0.1023958	608173	608173
13	0.04970167	292642	292642
14	0.01157971	70126	70126

ID# : 5
Name : 1,3 Propandiol
Quantitative Method : External Standard
Function : f(x)=5.53208e+006*x+60147.2
Rr1=0.9998524 Rr2=0.9997049 RSS=1.215679e+011
MeanRF: 5.669823e+006 RFSD: 1.091025e+005 RFRSD: 1.924266
FitType : Linear
ZeroThrough : Not Through
Weighted Regression : None
Offset Correction : Off
Detector Name : Detector A

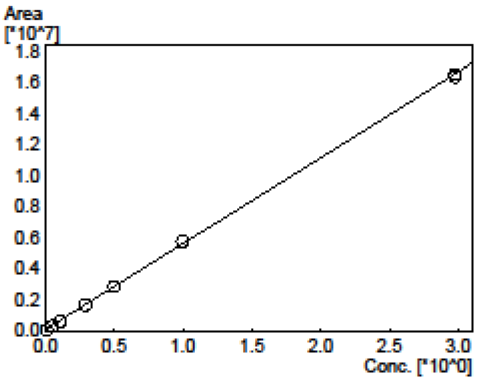

#	Conc.(Ratio)	MeanArea	Area
1	2.976375	16527734	16527734
2	0.9968958	5780232	5780232
3	0.4990984	2857708	2857708
4	0.2900131	1654742	1654742
5	0.10734	602395	602395
6	0.04944145	285398	285398
7	0.0100184	55116	55116
8	2.976375	16383858	16383858
9	0.9968958	5775464	5775464
10	0.4990984	2850795	2850795
11	0.2900131	1658624	1658624
12	0.10734	605236	605236
13	0.04944145	286442	286442
14	0.0100184	55579	55579

ID# : 6
Name : iso-Propanol
Quantitative Method : External Standard
Function : f(x)=3.79722e+006*x-22847.4
Rr1=0.9999788 Rr2=0.9999576 RSS=8.517058e+009
MeanRF: 3.693549e+006 RFSD: 9.671223e+004 RFRSD: 2.618409
FitType : Linear
ZeroThrough : Not Through
Weighted Regression : None
Offset Correction : Off
Detector Name : Detector A

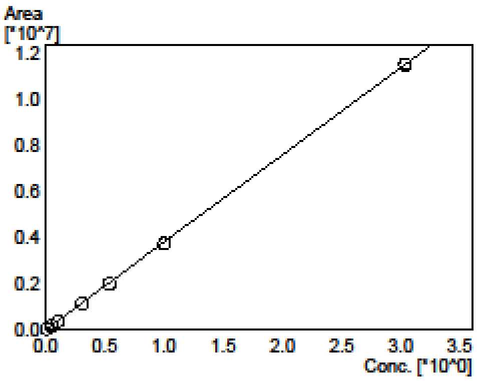

#	Conc.(Ratio)	MeanArea	Area
1	3.03666	11500380	11500380
2	1.003405	3731839	3731839
3	0.5418687	2008747	2008747
4	0.3101858	1147712	1147712
5	0.1067771	391809	391809
6	0.04972951	184593	184593
7	0.01397083	48408	48408
8	3.03666	11550446	11550446
9	1.003405	3755466	3755466
10	0.5418687	2022395	2022395
11	0.3101858	1155338	1155338
12	0.1067771	395336	395336
13	0.04972951	186359	186359
14	0.01397083	48851	48851

ID# : 7
Name : Hydroxyaceton
Quantitative Method : External Standard
Function : f(x)=4.86570e+006*x+58550.2
Rr1=0.9997691 Rr2=0.9995383 RSS=1.348973e+011
MeanRF: 5.021610e+006 RFSD: 2.667726e+005 RFRSD: 5.312492
FitType : Linear
ZeroThrough : Not Through
Weighted Regression : None
Offset Correction : Off
Detector Name : Detector A

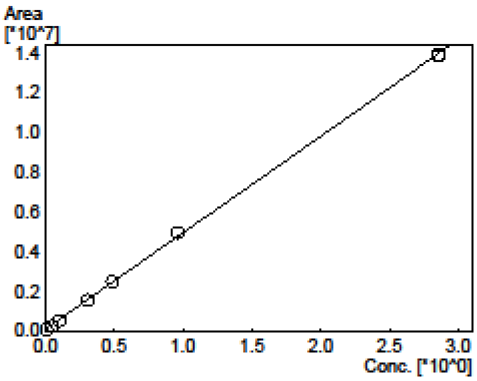

#	Conc.(Ratio)	MeanArea	Area
1	2.855514	13903987	14105284
			13702690
2	0.9609004	4949637	4949637
3	0.4875675	2469127	2469127
4	0.3072624	1547388	1547388
5	0.1038742	504414	504414
6	0.04860286	233159	233159
7	0.009361501	53654	53654
8	2.855514	13847720	14031978
			13663461
9	0.9609004	4959409	4959409
10	0.4875675	2471992	2471992
11	0.3072624	1546305	1546305
12	0.1038742	500893	500893
13	0.04860286	229656	229656
14	0.009361501	51129	51129

ID# : 8
Name : Ethylenglykol
Quantitative Method : External Standard
Function : f(x)=4.28481e+006*x+4449.72
Rr1=0.9999843 Rr2=0.9999685 RSS=7.572688e+009
MeanRF: 4.318958e+006 RFSD: 1.396465e+005 RFRSD: 3.233337
FitType : Linear
ZeroThrough : Not Through
Weighted Regression : None
Offset Correction : Off
Detector Name : Detector A

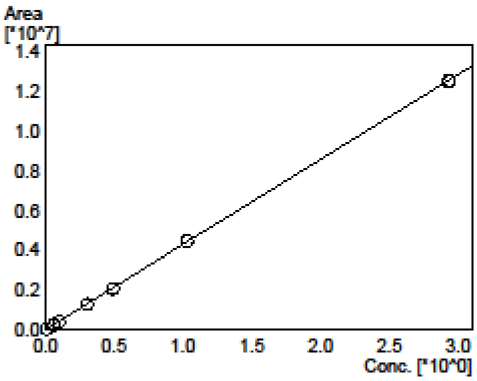

#	Conc.(Ratio)	MeanArea	Area
1	2.933794	12588577	12588577
2	1.030353	4453051	4453051
3	0.4898163	2091058	2091058
4	0.3033639	1293006	1293006
5	0.09840986	415192	415192
6	0.059971	250593	250593
7	0.01068632	49231	49231
8	2.933794	12532863	12532863
9	1.030353	4480928	4480928
10	0.4898163	2103205	2103205
11	0.3033639	1301865	1301865
12	0.09840986	417877	417877
13	0.059971	252466	252466
14	0.01068632	49741	49741

ID# : 9
Name : Ethanol
Quantitative Method : External Standard
Function : f(x)=2.10954e+006*x-9590.19
Rr1=0.9999717 Rr2=0.9999435 RSS=3.469230e+009
MeanRF: 2.228277e+006 RFSD: 4.145930e+005 RFRSD: 18.605995
FitType : Linear
ZeroThrough : Not Through
Weighted Regression : None
Offset Correction : Off
Detector Name : Detector A

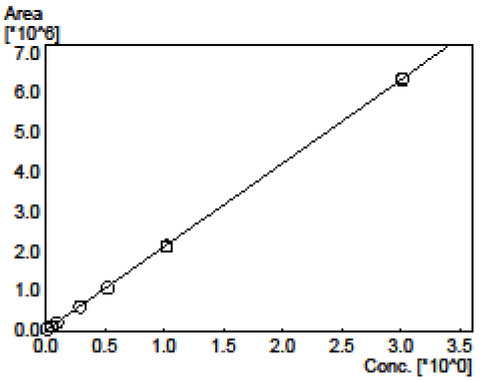

#	Conc.(Ratio)	MeanArea	Area
1	3.010594	6335586	6335586
2	1.02339	2122162	2122162
3	0.5257763	1079348	1079348
4	0.2939754	600851	600851
5	0.0961865	194742	194742
6	0.04960971	101619	101619
7	0.01299818	41467	41467
8	3.010594	6366832	6366832
9	1.02339	2137784	2137784
10	0.5257763	1087434	1087434
11	0.2939754	605513	605513
12	0.0961865	196464	196464
13	0.04960971	102375	102375
14	0.01299818	41854	41854

ID# : 10
Name : Propionsäure
Quantitative Method : External Standard
Function : f(x)=4.60145e+006*x-1609.00
Rr1=0.9999404 Rr2=0.9998807 RSS=3.533484e+009
MeanRF: 4.513152e+006 RFSD: 3.169498e+005 RFRSD: 7.022802
FitType : Linear
ZeroThrough : Not Through
Weighted Regression : None
Offset Correction : Off
Detector Name : Detector A

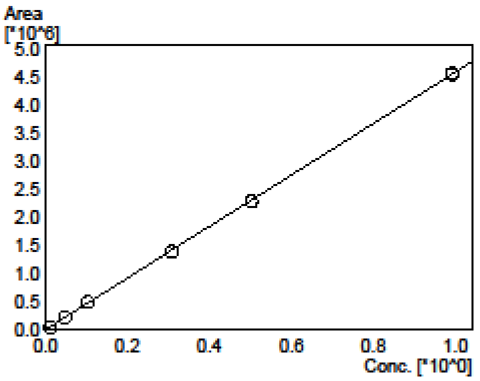

#	Conc.(Ratio)	MeanArea	Area
2	0.9915078	4559220	4559220
3	0.5019324	2284682	2284682
4	0.3085198	1394169	1394169
5	0.1036906	500724	500724
6	0.04835313	224032	224032
7	0.01168534	45098	45098
9	0.9915078	4584244	4584244
10	0.5019324	2300181	2300181
11	0.3085198	1403262	1403262
12	0.1036906	504256	504256
13	0.04835313	225438	225438
14	0.01168534	45425	45425

Figure S26: Calibration curves of all compounds detected by HPLC.

Retention time / min	Substance
0.69	Hydrogen
1.09	Oxygen
5.07	CO_2

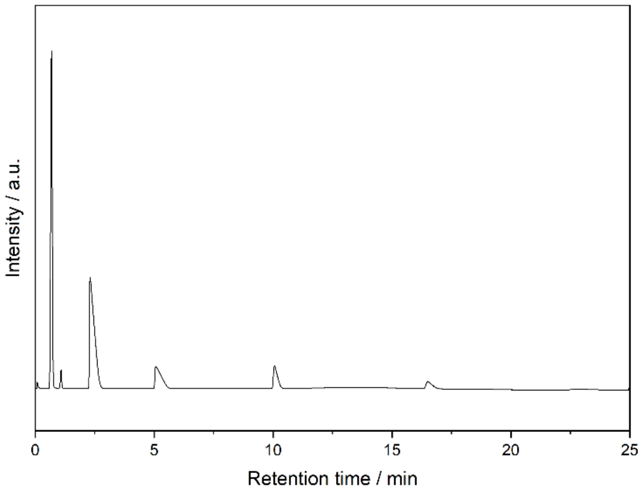

Figure S27: GC Chromatogram and retention times of the TCD detector of a usual gas sample.

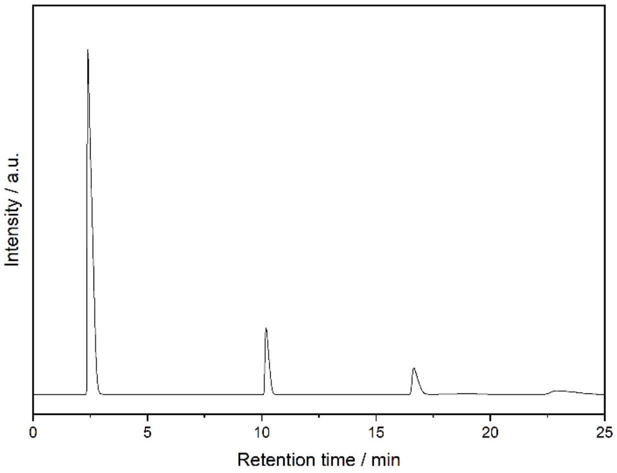

Retention time / min	Substance
2.40	Methane
10.20	Ethane
16.66	Propane
22.79	n-Butane

Figure S108: GC Chromatogram and retention times of the FID detector of a usual gas sample.

Table S7: Results of the glycerol hydrogenolysis experiments. $m_{support}$ = 100 mg, loading = 5wt%, T = 220 °C, p_{H2} = 30 bar, t = 20 h, n = 1000 rpm, (Gly) 20 wt. %, 10 g reaction mixture. 1,2-PDO = 1,2-propanediol, EG = Ethylene glycol, HA = Hydroxyaceton, n-Pr = n-Propanol, EtOH = Ethanol.

**Catalyst**	**Conversion / %**	**Selectivity / %**							**Carbon balance / %**
		1,2-PDO	EG	HA	n-Pr	EtOH	CH_4	C_2H_6	
NC7000	0.00	0.00	0.00	0.00	0.00	0.00	0.00	0.00	100.00
Baytubes	0.00	0.00	0.00	0.00	0.00	0.00	0.00	0.00	100.00
Flycarbon	0.00	0.00	0.00	0.00	0.00	0.00	0.00	0.00	100.00
TuBalls	0.00	0.00	0.00	0.00	0.00	0.00	0.00	0.00	100.00
Globugraphite	0.00	0.00	0.00	0.00	0.00	0.00	0.00	0.00	100.00
Ru-Cu_3/NC7000	59.71	40.53	6.72	4.11	4.19	2.56	2.80	0.33	91.78
Ru-Cu_3/Baytubes	59.11	74.61	5.18	2.98	3.18	2.69	0.90	0.10	92.31
Ru-Cu_3/Flycarbon	43.38	50.89	7.10	3.25	19.93	2.22	0.00	0.00	91.41
Ru-Cu_3/TuBalls	21.21	92.58	0.00	0.00	4.56	0.00	0.00	0.00	99.54
Ru-Cu_3/Globugraphite	74.89	56.27	3.65	9.60	11.61	3.65	0.00	0.00	86.78
Ru-Cu_5/Baytubes	56.96	67.15	4.86	3.61	11.91	2.43	0.85	0.55	92.88
Ru-Cu_4/Baytubes	52.68	74.15	5.05	2.80	9.17	2.01	1.65	0.35	96.22
Ru-Cu_2/Baytubes	70.89	56.68	5.50	7.20	8.96	3.80	3.48	1.10	88.47
Ru-Cu/Baytubes	80.01	48.97	0.06	10.55	9.22	5.22	0.00	0.00	77.82
Ru_2-Cu/Baytubes	86.35	48.56	5.07	8.06	6.97	3.28	1.49	0.30	63.44
Ru_3-Cu/Baytubes	87.86	48.48	3.51	7.10	8.12	4.07	2.60	0.30	75.27
Ru-Cu_3/Baytubes (N)	67.53	67.10	5.11	3.93	8.32	2.27	6.23	1.13	95.58
Ru-Cu_3/Baytubes (Cl)	64.87	64.62	4.27	5.08	8.44	2.55	1.73	0.57	91.33
Ru-Cu_3/Baytubes (OAc)	79.43	46.49	4.11	10.44	9.83	5.55	0.00	0.00	80.77
Ru-Cu_3/Baytubes (acac)	50.63	76.87	3.90	3.99	10.63	2.30	0.83	0.13	98.45
Ru-Cu_3/Baytubes-IWI-H_2O	75.26	47.93	4.08	10.08	8.23	4.41	3.90	1.05	84.55
Ru-Cu_3/Baytubes-IWI-EtOH	69.30	52.18	5.08	10.24	7.53	3.37	1.50	0.55	84.83
Ru-Cu_3/Baytubes-CD (OR)	55.66	62.72	4.63	5.79	10.86	2.70	2.20	1.30	93.26
RuCu_3/Baytubes-CD (CR)	60.12	54.42	4.13	9.01	11.12	3.99	3.10	1.23	91.25
Ru-Cu_3/Baytubes-DP	11.75	88.74	0.00	2.04	11.59	0.00	0.00	0.00	100.14
Ru-Cu_3/Baytubes-CP	69.53	62.88	3.26	7.80	9.69	2.79	0.00	0.00	89.81
Ru-Cu_3/Baytubes-SSG	59.72	65.80	6.75	7.76	7.20	2.30	0.00	0.00	91.80
Ru-Cu_3/Baytubes-BMSSG	34.88	81.57	5.20	2.20	9.46	0.00	0.00	0.00	98.86

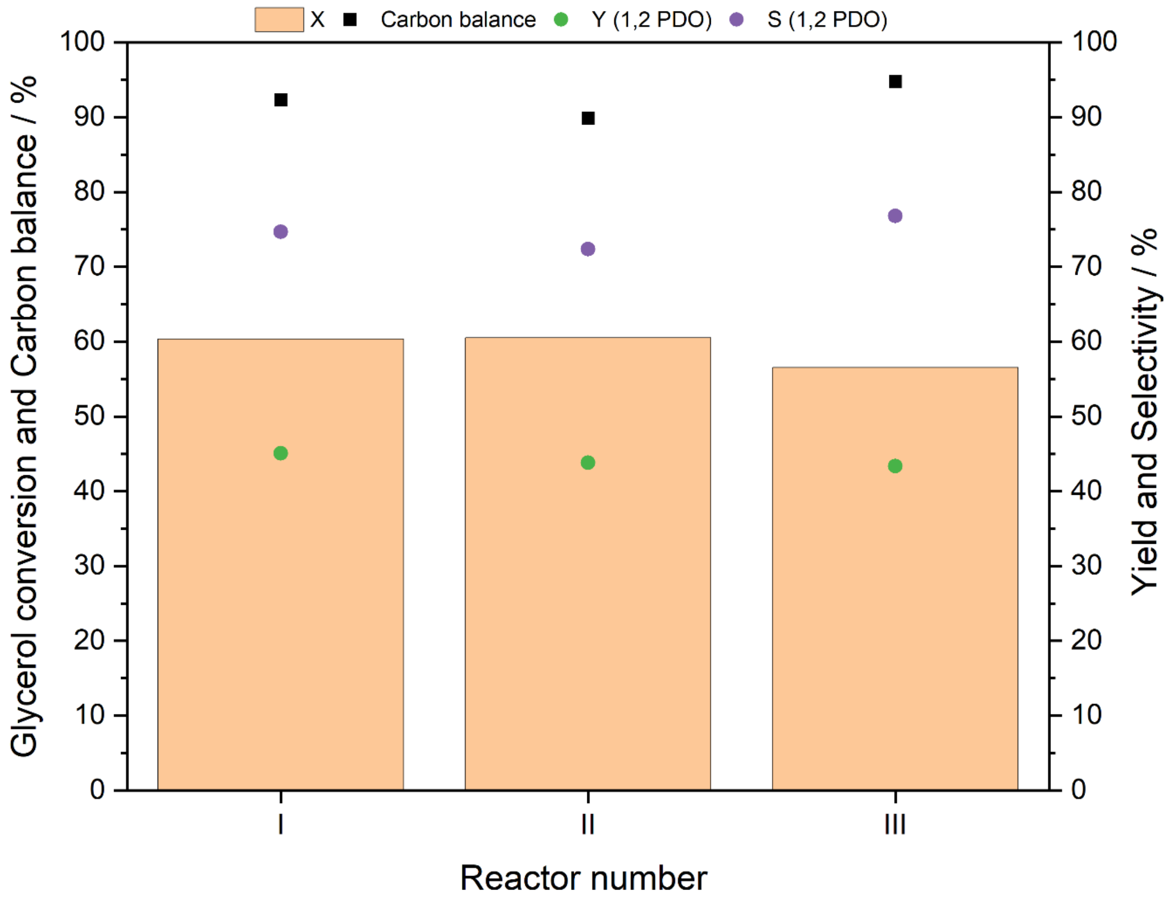

Figure S29: Reproducibility study of the Ru-Cu_3/Baytubes catalyst. m_{Cat} = 100mg, loading = 5wt%, T = 220 °C, pH_2 30 bar, t = 20 h, n = 1000 rpm, (Gly) 20 wt. %, 10 g reaction mixture.

The reproduction study gives us a standard deviation for the conversion of 1.85 %, 0.72 % for the yield, 1.80 % for selectivity and 1.98 % for the carbon balance. These values are taken as standard derivation, if an experiment wasn't repeated.

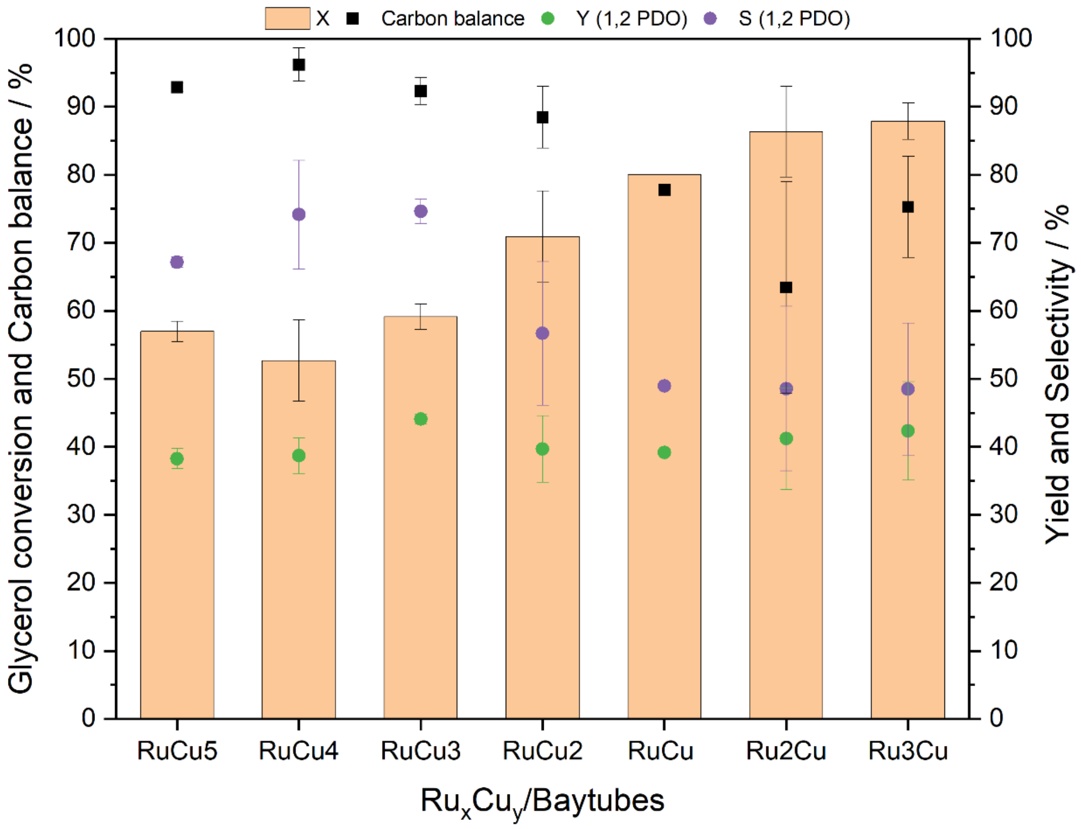

Figure S30: Catalytic performance of the catalyst with different Ru-Cu ratio supported on Baytubes. m_{Cat} = 100 mg, loading = 5 wt. %, T = 220 °C, p_{H2} 30 bar, t = 20 h, n = 1000 rpm, (Gly) 20 wt.%, 10 g reaction mixture.

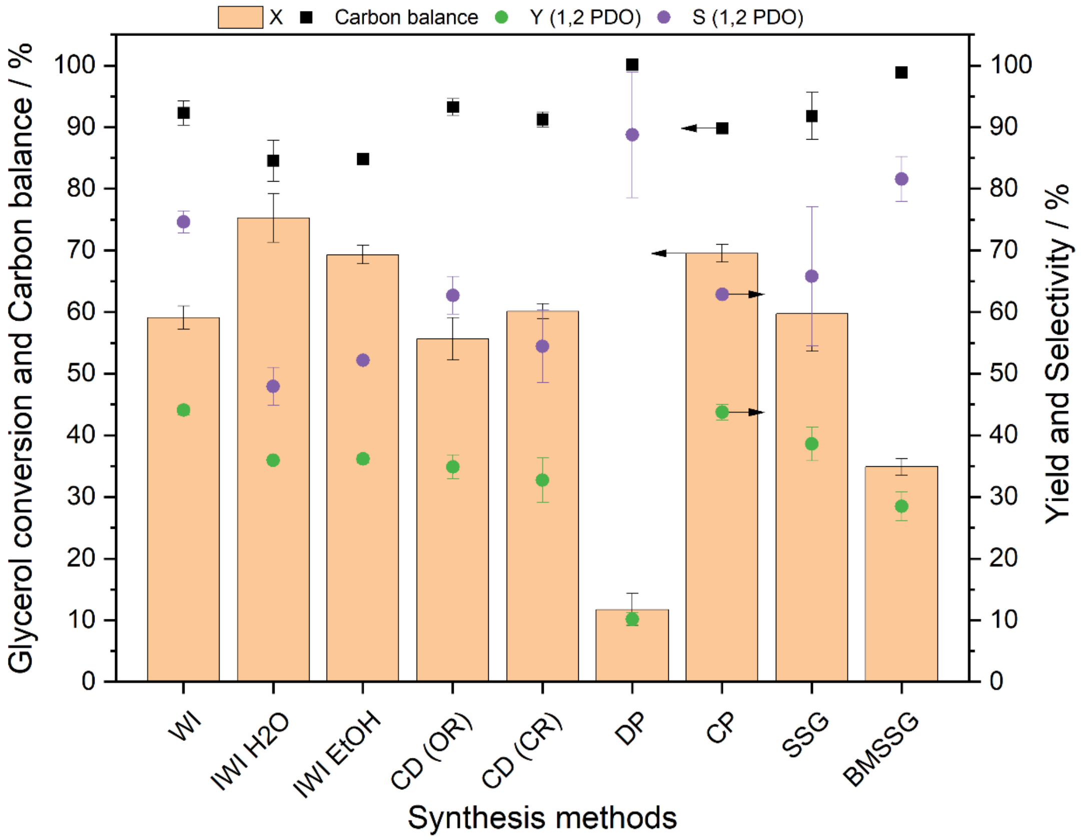

Figure S31: Catalytic performance of different Ru-Cu_3/Baytubes catalysts, were different precursors have been used. WI = wetness impregnation, IWI = incipient wetness impregnation, CD = chemical deposition, DP = deposition precipitation, CP = co precipitation, SSG = solid state grinding, BMSSG = ball mill solid state grinding. m_{Cat} = 100 mg, loading = 5 wt. %, T = 220 °C, p_{H2} 30 bar, t = 20 h, n = 1000 rpm, (Gly) 20 wt.%, 10 g reaction mixture.

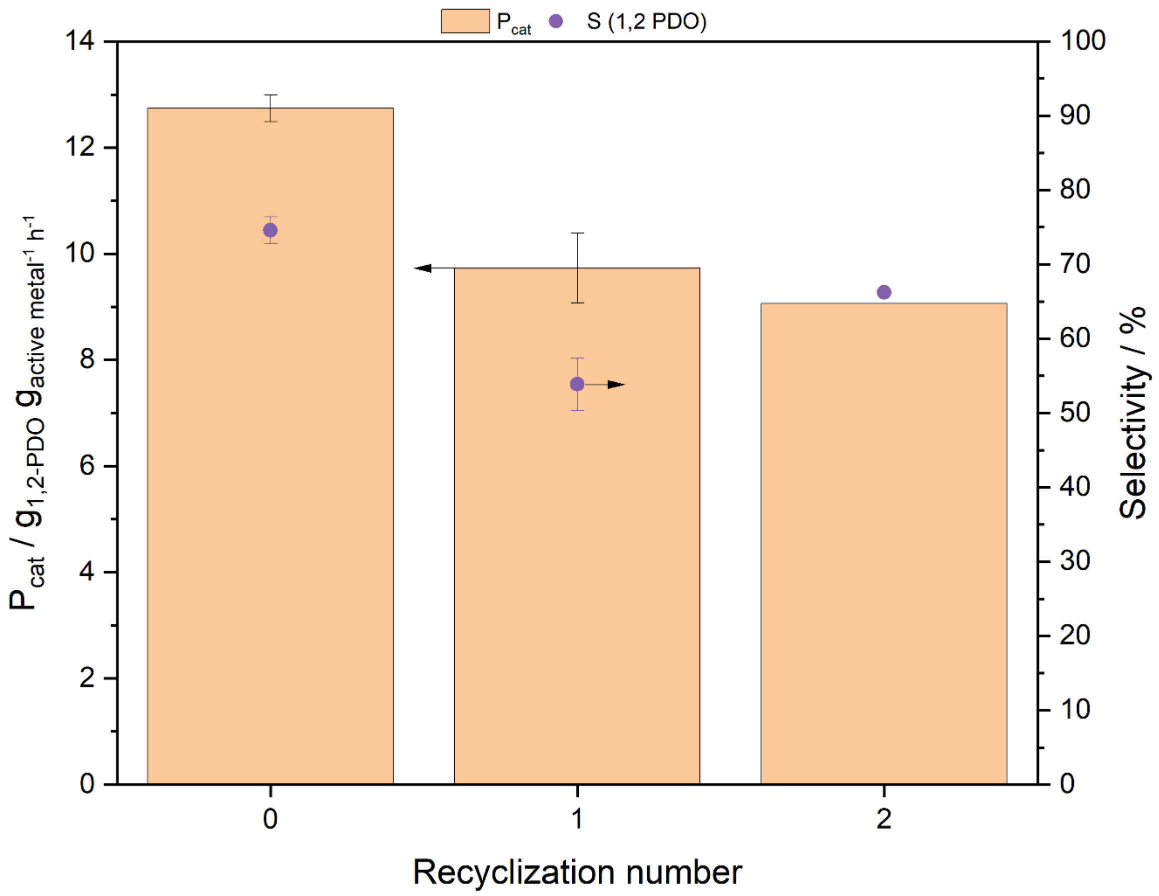

Figure S32: Recycling study of the Ru-Cu_3/Baytubes catalyst. m_{Cat} = 100 mg, loading = 5 wt. %, T = 220 °C, p_{H2} 30 bar, t = 20 h, n = 1000 rpm, (Gly) 20 wt.%, 10 g reaction mixture.

Table S8: Comparison of the catalyst activity with key literature results.

**Reference**	**Catalyst composition**	**Productivity/ $g_{1,2\text{-PDO}}$ $g_{\text{active metal}}^{-1}$ h^{-1}**	**Reaction conditions**
This work	$RuCu_3$/Baytubes	12.74±0.25	T = 220 °C, p = 30 bar H_2, *20 wt % Glycerol, 10 ml reaction solution*, m_{cat} = 100 mg, 5% metal loading, t = 20 h
Lumpp et al.[1]	$RuCu_3$/NC7000	9.79±0.00	T = 220 °C, p = 30 bar H_2, *20 wt % Glycerol, 10 ml reaction solution*, m_{cat} = 100 mg, 5% metal loading, t = 20 h
Sherbi et al.[2]	$RuCu_2$/NC7000	1.38	T = 200 °C, p = 35 bar H_2, *20 wt % Glycerol, 10 ml reaction solution,* m_{cat} = 200 mg, 5% metal loading, t = 20 h
Li et al.[3]	Ru_2Fe/CNT	TOF / h^{-1} = 147.5	T = 200 °C, p = 40 bar H_2, *20 wt % Glycerol, 20 ml reaction solution,* m_{cat} = 250 mg, 5% metal loading, t = 12 h
Wu et al.[4]	CuRu/CNT	TOR at 5% conversion / (10^{-3} mol $mol^{-1}_{\text{surface-metal}}$ s^{-1} = 3.9 (Cu)	T = 200 °C, p = 40 bar H_2, *80 wt % Glycerol, 20 ml reaction solution,* m_{cat} = 800 mg, 10 % metal loading, t = 6 h
Miyazawa et al.[5]	Ru/C+Amberlyst	1.63	T = 120 °C, p = 80 bar H_2, *20 wt % Glycerol, 20 ml reaction solution,* m_{cat} = 150 mg + 300 mg Amberlyst, 5 % metal loading, t = 10 h

References

(1) Lumpp, D.; Shaikh, S.; Riebesehl, F.; Ruhmlieb, C.; Kruber, K.; Schroeter, B.; Smirnova, I.; Skiborowski, M.; Fiedler, B.; Albert, J. Multifunctional Carbon-Nanotube Supported Catalyst for Efficient Glycerol Hydrogenolysis to 1,2-Propanediol. *ChemCatChem*.

(2) Sherbi, M.; Wesner, A.; Wisniewski, V. K.; Bukowski, A.; Velichkova, H.; Fiedler, B.; Albert, J. Superior CNT-Supported Bimetallic RuCu Catalyst for the Highly Selective Hydrogenolysis of Glycerol to 1,2-Propanediol. *Catal. Sci. Technol.* **2021**, *11* (20), 6649–6653. https://doi.org/10.1039/d1cy01518d.

(3) Li, B.; Wang, J.; Yuan, Y.; Ariga, H.; Takakusagi, S.; Asakura, K. Carbon Nanotube-Supported RuFe Bimetallic Nanoparticles as Efficient and Robust Catalysts for Aqueous-Phase Selective Hydrogenolysis of Glycerol to Glycols. *ACS Catal.* **2011**, *1* (11), 1521–1528. https://doi.org/10.1021/cs200386q.

(4) Wu, Z.; Mao, Y.; Wang, X.; Zhang, M. Preparation of a Cu–Ru/Carbon Nanotube Catalyst for Hydrogenolysis of Glycerol to 1,2-Propanediol via Hydrogen Spillover. *Green Chem.* **2011**, *13* (5), 1311–1316. https://doi.org/10.1039/c0gc00809e.

(5) Miyazawa, T.; Kusunoki, Y.; Kunimori, K.; Tomishige, K. Glycerol Conversion in the Aqueous Solution under Hydrogen over Ru/C + an Ion-Exchange Resin and Its Reaction Mechanism. *J. Catal.* **2006**, *240* (2), 213–221. https://doi.org/10.1016/j.jcat.2006.03.023.
